# Neutrophil‐Mimetic Nanoscavengers Target the Inflammatory Microenvironment to Eliminate NETs/ROS and Immunomodulate cGAS‐STING Signaling in Septic AKI

**DOI:** 10.1002/advs.202521861

**Published:** 2026-02-15

**Authors:** Zening Zhang, Chenxi Zhang, Ranran Luo, Qiuchi Wu, Pengchen Ren, Xinyu Liu, Yingying Luo, Zhongsheng Xu, Xiaojing He, Yun Liu

**Affiliations:** ^1^ Department of Radiology The Second Affiliated Hospital of Chongqing Medical University Chongqing China

**Keywords:** cGAS‐STING inhibition, nanoscavenger, NETs degradation, ROS scavenging, septic acute kidney injury

## Abstract

Sepsis‐associated acute kidney injury (SAKI) remains a life‐threatening condition with limited therapeutic options, primarily driven by rampant oxidative stress, inflammatory dysregulation. Importantly, aberrant formation of neutrophil extracellular traps (NETs) and sustained innate immune activation further exacerbate renal injury, highlighting the need for strategies that precisely modulate these intertwined pathological mechanisms. Here, we present neutrophil‐mimetic nanoscavengers (MD@NM) that comprise a catalytic core of DNase‐1‐loaded Mn_3_O_4_ nanozymes enveloped by a neutrophil membrane engineered to actively target and simultaneously disrupt multiple pathological circuits in SAKI. The neutrophil membrane confers chemokine‐receptor (e.g., CXCR2) mediated homing to injured kidneys, while the Mn_3_O_4_ nanozymes catalytically scavenge ROS and the loaded DNase‐1 enzymatically degrades NETs‐derived extracellular DNA, thereby suppressing the cGAS‐STING pathway and skewing macrophage polarization toward an M2 reparative phenotype. In a murine model of LPS‐induced SAKI, MD@NM treatment facilitated robust renal targeting, attenuated neutrophilic infiltration, resolved cytokine storm, and ameliorated structural kidney damage. Collectively, this biomimetic platform represents a novel strategy for precision immunomodulation and multi‐mechanistic therapy against SAKI by integrating antioxidative, NETs‐scavenging, and anti‐inflammatory functions into a single nanotherapeutic agent.

## Graphical abstract

Sepsis‐associated acute kidney injury is driven by oxidative stress, NETs overproduction, and dysregulated innate immunity. Here, we develop neutrophil‐mimetic nanoscavengers that integrate ROS‐scavenging Mn_3_O_4_ nanozymes with DNase‐1‐mediated NETs degradation to suppress cGAS‐STING signaling, reprogram macrophage polarization, and effectively alleviate renal inflammation and injury in septic AKI.

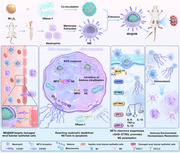

## 1 Introduction

Acute kidney injury (AKI) is a common and severe manifestation of organ dysfunction, affecting 5% of hospitalized patients [1] and up to 50% of those in intensive care units (ICUs) [2, 3]. Notably, sepsis remains the leading cause of AKI in critically ill patients [3, 4], and when AKI occurs as part of sepsis‐associated multiple organ dysfunction syndrome (MODS), mortality exceeds 50%, reflecting its profound clinical burden [5, 6]. Despite its critical clinical significance, the pathophysiological mechanisms driving septic AKI remain incompletely elucidated. Nevertheless, even as the full spectrum of these mechanisms remains to be fully delineated, accumulating evidence demonstrates that immune dysregulation and unbridled inflammatory cascades exert pivotal roles in the initiation and progression of infection‐induced AKI, thus positioning inflammation and immunomodulation as promising targets for therapeutic intervention [5, 7].

During sepsis, invading bacteria release substantial quantities of lipopolysaccharide (LPS) [8], which acts as a toxin that provokes intense inflammation and oxidative stress [9, 10, 11], two pathological processes central to the development of SAKI [12]. These cascades trigger excessive production of reactive oxygen species (ROS), ultimately leading to widespread cellular dysfunction and severe renal tissue injury [13, 14, 15]. Although conventional antioxidant small molecules and natural enzymes have been clinically explored for therapeutic intervention, their utility remains limited by poor stability, inadequate catalytic activity [16], and rapid inactivation under the persistent oxidative stress associated with sepsis [17]. Recent breakthroughs in nanomedicine have enabled the development of nanozymes, nanomaterials with intrinsic enzyme‐mimetic activities, including CeO_2_, Mn_3_O_4_, Fe_3_O_4_, Pt, and Au [18]. Compared with biological enzymes, these nanozymes exhibit broad‐spectrum ROS‐scavenging capabilities and superior catalytic durability, which allows rapid and efficient ROS elimination to slow or halt the progression of AKI [19, 20, 21]. Among these nanozymes, ultrasmall Mn_3_O_4_‐based systems are particularly attractive due to their redox versatility, multi‐enzyme mimicry, and excellent biocompatibility [22, 23], and the essential physiological role of manganese further enhances their translational potential [24]. Nevertheless, mounting evidence indicates that ROS scavenging alone is insufficient to fully reverse SAKI [13, 25], highlighting the urgent need for more precise and multi‐targeted nanotherapeutic strategies to treat this complex condition.

Within the inflammatory microenvironment, neutrophils, the most prevalent innate immune cells in the bloodstream [26], are swiftly recruited to the damaged organs, including arthritis, colitis, sepsis, acute lung injury and liver failure [27]. They initially release proinflammatory mediators that amplify local inflammation [28], and upon persistent stimulation during the ensuing cytokine storm, undergo NETosis characterized by the extrusion of double‐stranded DNA to form neutrophil extracellular traps (NETs). NETs, formed by the extrusion of chromatin fibers ornamented with histones and proteolytic enzymes, are increasingly recognized as critical mediators of inflammatory responses [28, 29, 30, 31, 32, 33]. Neutrophil activation induced by NETs occurs through multiple intracellular signaling cascades, including the phosphorylation of Akt, ERK1/2, and p38 [34]. Moreover, evidence indicates that the DNA scaffolds of NETs can activate the cytosolic DNA sensor cGAS, thereby initiating the cGAS‐STING signaling pathway [35]. This cascade elicits robust production of type I interferons and proinflammatory cytokines, thereby aggravating the inflammatory milieu. Such excessive signaling establishes a self‐perpetuating vicious cycle of tissue injury. Together, these insights identify NETs as pivotal regulators of inflammatory cascades and highlight them as promising therapeutic targets in NETs‐associated disorders.

However, studies of SAKI have traditionally emphasized the roles of cytokine storms, macrophage dysregulation, and endothelial dysfunction, while the involvement of NETs has received comparatively less attention. Whether NETs make a decisive contribution to the immunopathology of SAKI remains insufficiently understood, thus raising the possibility that NETs may represent an underappreciated component of SAKI pathogenesis. Among various strategies to dismantle NETs, deoxyribonuclease I (DNase‐1) has emerged as a particularly promising candidate [36, 37]. DNase‐1 is a ubiquitous endonuclease capable of cleaving both single‐ and double‐stranded DNA, widely distributed in vertebrate serum and tissues (e.g., bovine pancreas), and has been applied in diverse biomedical contexts [37]. Although multiple nucleases, such as DNase II, possess DNA hydrolytic activity, they differ substantially in their site of action, translational feasibility, and safety profiles. DNase‐II predominantly localizes to lysosomes and exhibits optimal activity under acidic conditions, rendering it less effective for degrading extracellular NETs‐derived DNA in the neutral physiological milieu [38]. By comparison, DNase‐1 is a classical extracellular endonuclease that has been extensively employed in NETs clearance‐related studies and engineered delivery systems. It is widely regarded as a representative and clinically relevant candidate for NETs degradation, with a more established translational foundation and superior compatibility for therapeutic engineering [39, 40, 41]. Its unique advantage lies in efficiently degrading the DNA backbone of NETs, thereby preventing their persistence and mitigating downstream activation of the cGAS‐STING pathway [42]. This process not only reduces NETs‐mediated structural tissue damage but also alleviates excessive immune activation. Consequently, strategies that leverage DNase‐1 for NETs elimination represent a promising avenue to immunomodulate the cGAS‐STING pathway and restore immune balance in SAKI.

**SCHEME 1:**
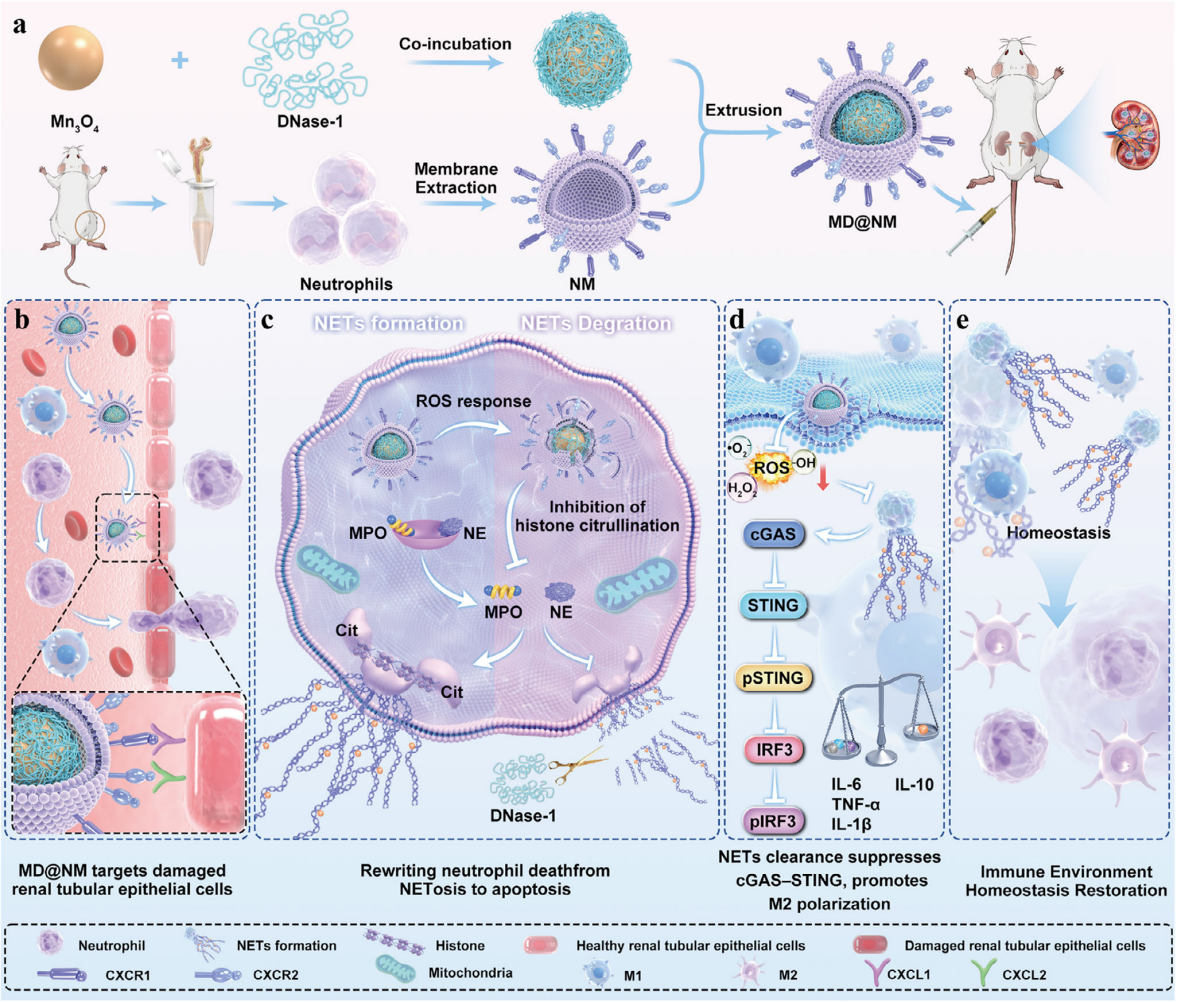
Schematic illustration of neutrophil‐mimetic nanoscavengers for the treatment of SAKI. (a) Preparation of DNase‐1‐loaded Mn_3_O_4_ nanozymes (MD NPs) and neutrophil membrane coating to form MD@NM nanoscavengers; (b) Chemokine CXCL1/2 mediates the recognition and adhesion of MD@NM to inflamed renal tissue, achieving precise accumulation; (c‐e) In the inflammatory microenvironment, MD@NM scavenges ROS, degrades NETs, suppresses cGAS‐STING signaling, and promotes macrophage M2 polarization, collectively mitigating inflammation and restoring renal homeostasis.

Here, we performed transcriptomic analyses of patient samples and mouse models, including GSE232404 and kidney RNA sequencing, and discovered a pronounced enrichment of NETs‐related signatures along with persistent activation of the cGAS‐STING signaling pathway. These changes were accompanied by upregulation of inflammatory mediators, reflecting sustained neutrophil activation and ongoing innate immune stimulation. Further network analysis revealed that extracellular DNA released from NETs acts as a key driver of cGAS‐STING signaling, amplifying interferon responses and exacerbating renal injury. Collectively, these findings highlight the central role of the NETs‐cGAS‐STING axis in the immunopathology of SAKI and underscore its therapeutic relevance. Inspired by these insights, we developed a multifunctional neutrophil‐mimetic nanoscavenger platform in which DNase‐1 was loaded onto Mn_3_O_4_ nanozymes (MD NPs) and subsequently camouflaged with neutrophil membranes to form MD@NM nanoscavengers (Scheme 1a). The Mn_3_O_4_ core confers potent redox activity for efficient ROS elimination [43], whereas the neutrophil membrane coating imparts inflammatory homing and immune evasion [44, 45, 46], while simultaneously stabilizing DNase‐1 activity to enable efficient NETs degradation [33]. In the inflammatory microenvironment of renal tissues, elevated chemokines such as CXCL1 and CXCL2 [47], promote the adhesion and precise accumulation of MD@NM nanoscavengers (Scheme 1b). Within the inflammatory microenvironment, MD@NM nanoscavengers not only scavenge excessive ROS but also degrade the DNA scaffold of NETs, thereby reducing pathological dsDNA exposure, suppressing cGAS‐STING activation, and reprogramming macrophages toward an anti‐inflammatory M2 phenotype (Scheme 1c‐e). This dual reprogramming of NETs and macrophages, together with robust ROS elimination, effectively mitigates the inflammatory cascade of SAKI, restores the renal microenvironment, and provides a precise and multifunctional therapeutic strategy to counteract sepsis‐associated acute kidney injury.

## 2 Results and Discussion

### 2.1 Transcriptomic Insight Into NETs and Inflammatory Pathways Driving SAKI

**FIGURE 1:**
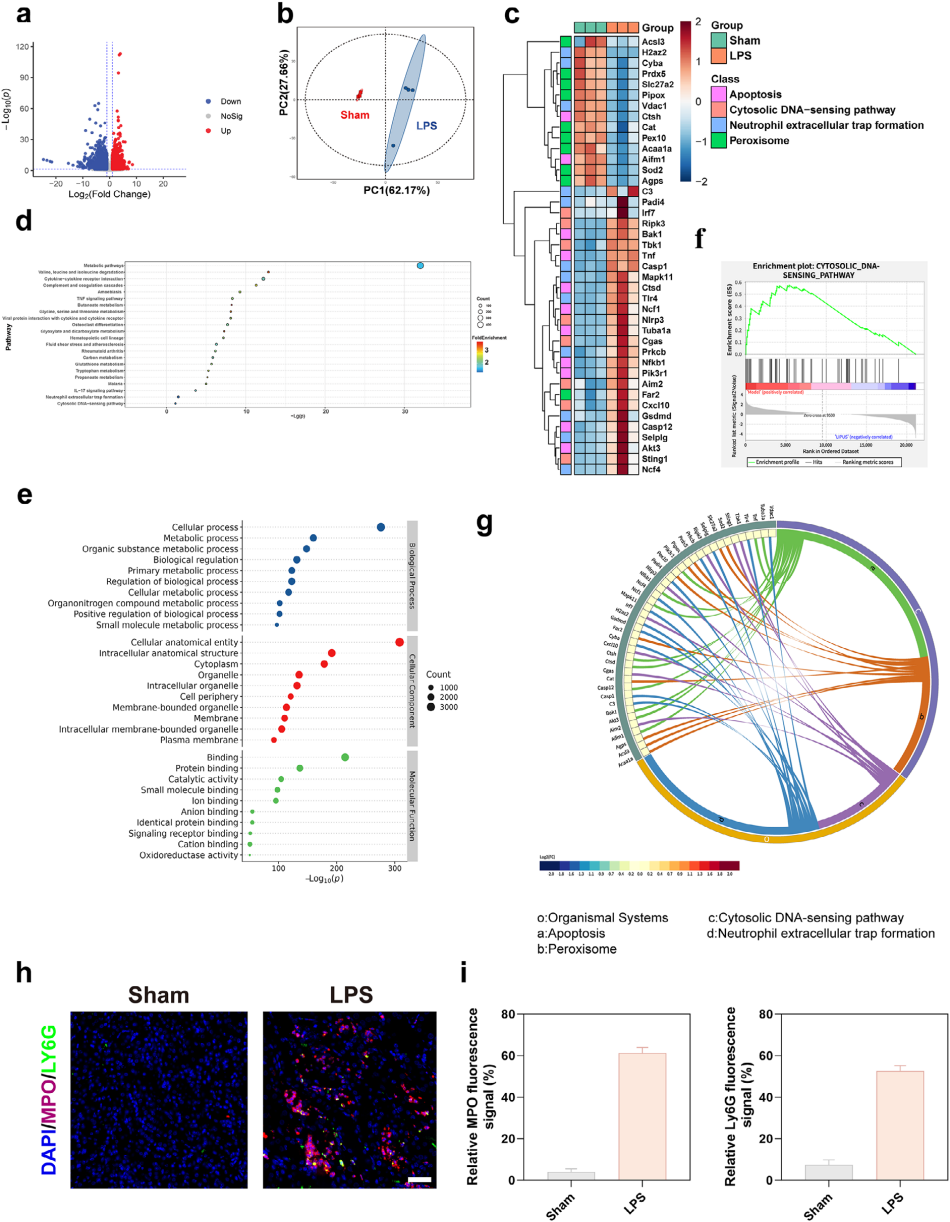
Transcriptomic insight into NETs and inflammatory pathways driving SAKI. (a) Volcano plot illustrating DEGs between the Sham and PBS control groups in renal tissues. (b) PCA of RNA‐Seq data reveals distinct transcriptomic profiles among different groups. (c) Heatmap showing gene expression patterns across groups based on mRNA sequencing data. (d) KEGG pathway enrichment analysis of the top 22 significantly altered genes between Sham and LPS groups. (e) GO enrichment analysis of the top 10 DEGs between the Sham and LPS groups. (f) GSEA identifying activation of the cGAS‐STING signaling pathway in LPS versus Sham group. (g) Combined KEGG‐GO chord plot highlighting key biological processes and pathways involved in sepsis‐induced AKI pathogenesis. (h) Representative immunofluorescence images showing Ly6G‐positive neutrophils (green) and H3Cit‐positive NETs (red); nuclei are counterstained with DAPI (blue). Scale bar: 50 µm. (i) Quantitative analysis of the relative fluorescence intensity of MPO and H3Cit (n = 3). Data are shown as the mean values ± SD (n = 3). Statistical significance was determined by ANOVA. ^*^ *p* < 0.05, ^**^ *p* < 0.01, ^***^ *p* < 0.001; ns, not significant.

To evaluate the clinical relevance of NETs and neutrophil‐mediated inflammation in SAKI, we first analyzed publicly available transcriptomic datasets from SAKI patients in the Gene Expression Omnibus (GEO). Compared with healthy controls, patients with SAKI exhibited significantly higher NETs formation scores, indicating aberrant NETs activation in vivo (Figure S1). Concurrently, immune cell infiltration analysis revealed markedly elevated neutrophil activation in the SAKI group, reflecting a hyperinflammatory immune profile (Figure S2). These clinical data underscore the pathogenic contribution of NETs and neutrophil‐driven immune dysregulation in SAKI, emphasizing the urgent need for targeted interventions. To validate these findings in an animal model, we performed whole‐transcriptome sequencing of renal tissues from sham group and LPS group mice. This enabled comprehensive mechanistic investigation of the disease and therapeutic response. Differential gene expression analysis identified substantial numbers of differentially expressed genes (DEGs) between the LPS group and sham group (Figure 1a). Principal component analysis (PCA) demonstrated distinct clustering of LPS samples away from sham controls (Figure 1b), indicative of a robust inflammatory shift in the SAKI model. Heatmap analysis further highlighted the dysregulation of genes related to NETs, redox enzymes, apoptosis, and innate immunity in LPS group kidneys (Figure 1c). Gene Ontology (GO) and Kyoto Encyclopedia of Genes and Genomes (KEGG) enrichment analyses revealed that pathways associated with NETs formation, DNA sensing, and the cGAS‐STING signaling cascade were significantly activated in LPS group kidneys (Figure 1d,e). These results suggest that NETs, through the release of extracellular DNA, may potentiate inflammation via activation of innate DNA‐sensing pathways, particularly the cGAS‐STING axis. KEGG enrichment of the top 22 DEGs underscored the involvement of STING signaling, inflammatory cytokine production, and neutrophil activation (Figure 1f). In addition, chord plots integrating KEGG and GO data revealed a strong interconnection between these pathways, reinforcing the pivotal roles of NETs and cGAS‐STING signaling in SAKI progression (Figure 1g). Among them, the cGAS‐STING axis emerged as a critical mediator of inflammation in SAKI, given its ability to detect cytosolic dsDNA and initiate downstream immune activation [48, 49]. NETs formation facilitates this process by releasing nuclear DNA into the extracellular space, thereby exacerbating inflammatory responses [26]. The transcriptomic findings were further corroborated by immunofluorescence analysis, which showed extensive NETs accumulation in LPS group renal tissues, while NETs were virtually absent in the sham group (Figure 1h,i). Collectively, these results reveal that aberrant NETs formation and overactivation of the cGAS‐STING pathway constitute central pathological events in SAKI, providing a mechanistic rationale for subsequent targeted therapeutic strategies.

### 2.2 Preparation and Characterizations of MD@NM Nanoscavengers

**FIGURE 2:**
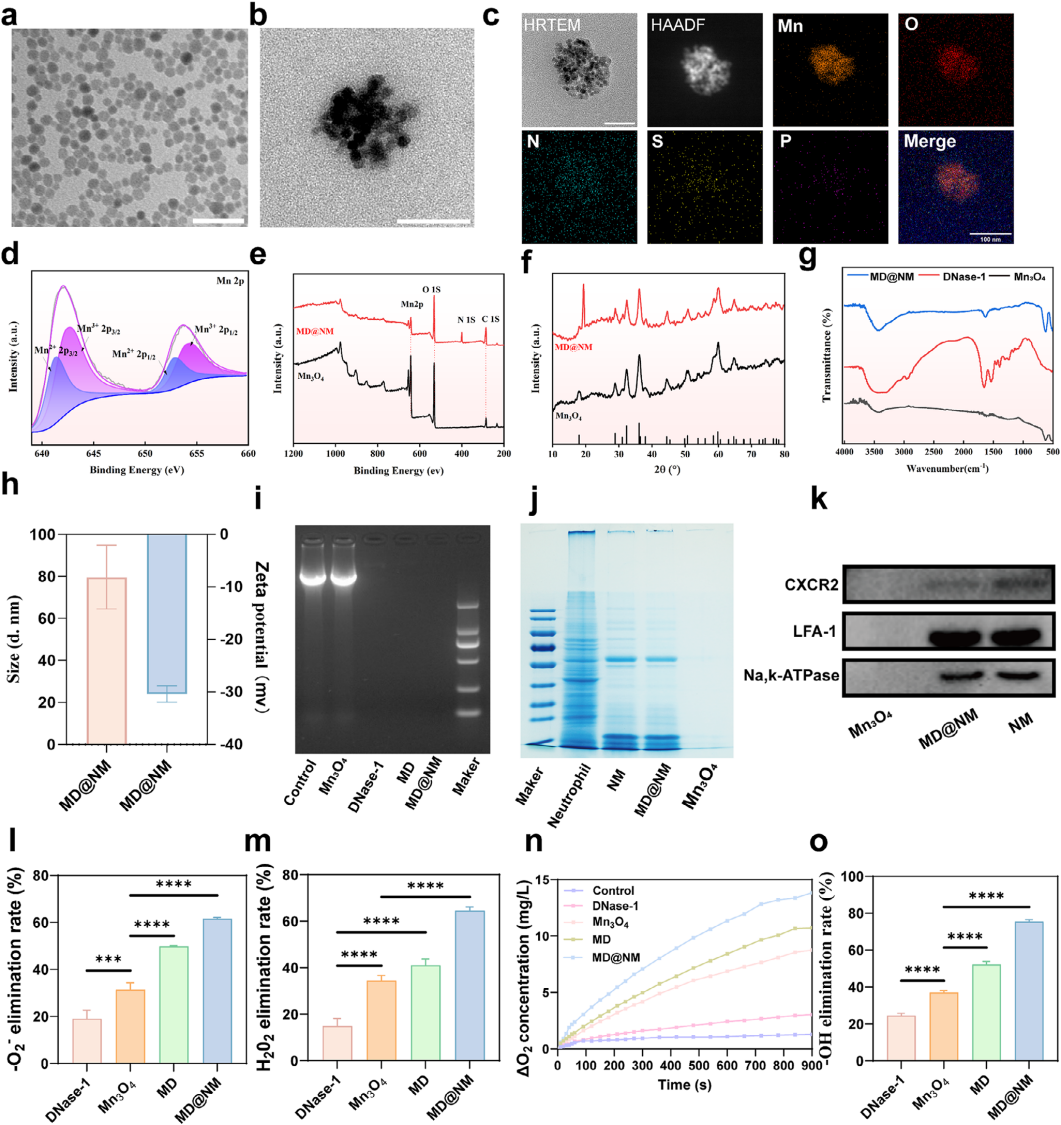
Characterization of MD@NM nanoscavengers: (a) TEM image of the synthesized Mn_3_O_4_ nanozymes. Scale bar: 50 nm. (b) TEM image of MD@NM nanoscavengers. Scale bar: 50 nm. (c) Elemental mapping of MD@NM. Scale bar: 50 nm. (d‐e) XPS spectra of Mn 2p and survey scan for Mn_3_O_4_ and MD@NM. (f) XRD patterns of Mn_3_O_4_ and MD@NM. (g) FTIR spectra of Mn_3_O_4_, DNase‐1, and MD@NM. (h) zeta potential and hydrodynamic size distribution of MD@NM. (i) Agarose gel electrophoresis of DNA after treatment with different formulations. (j) SDS‐PAGE analysis of protein components from neutrophils, neutrophil membranes (NM), MD, and MD@NM. (k) Western blot analysis of chemotaxis‐ and adhesion‐related membrane proteins in Mn_3_O_4_, NM, and MD@NM, verifying membrane protein retention. (l‐o) Evaluation of the ROS‐scavenging capability of MD@NM, including SOD‐like activity (l), CAT‐like activity (m), decomposition of H_2_O_2_ to release O_2_ (n), and hydroxyl radical (•OH) scavenging ability (o). The data are presented as the means ± SD (n = 3). Statistical significance was determined by ANOVA. ^*^ *p* < 0.05, ^**^ *p* < 0.01, ^***^ *p* < 0.001; ns, not significant.

Based on transcriptomic and immune infiltration analyses indicating excessive neutrophil activation and NETs formation in septic AKI, we designed a multifunctional biomimetic nanoscavengers system to support the therapeutic mechanism proposed above. Specifically, we constructed a neutrophil‐mimetic nanoscavengers (MD@NM) composed of a Mn_3_O_4_ core, DNase‐1 conjugation, and a neutrophil membrane shell, with the aim of targeting inflamed renal tissues and modulating the immune microenvironment. Mn_3_O_4_ were first synthesized via a hydrothermal method [50]. Transmission electron microscopy (TEM) showed that the resulting Mn_3_O_4_ nanoscavengers were monodispersed and exhibited a uniform spherical morphology (Figure 2a), with a hydrodynamic diameter of 9.58 ± 1.81 nm (Figure S3). Following the loading of DNase‐1, the zeta potential of the nanoparticles shifted markedly from +3.74 to −24.39 mV (Figure S7), indicating successful surface modification. Subsequent coating with neutrophil membranes resulted in the formation of MD@NM nanoscavengers, which retained a well‐defined core‐shell architecture, with a clearly distinguishable outer membrane layer observable by TEM (Figure 2b), and the hydrodynamic diameter increased to 62.77 nm. Elemental mapping confirmed the uniform distribution of Mn, O, N, P, and S elements on the particle surface (Figure 2c), supporting the successful integration of enzyme and membrane components. Mn_3_O_4_ possesses two redox‐active manganese valence states [51]. Meanwhile, the zeta potential shifted from −24.39 to −30.38 mV, indicating successful coating with negatively charged neutrophil membranes (Figure 2h). X‐ray photoelectron spectroscopy (XPS) analysis showed that MD@NM nanoscavengers retained the characteristic Mn 2p peaks of Mn_3_O_4_, with Mn 2p^3/2^ centered at 641.01 and 642.35 eV, corresponding to Mn^2+^ and Mn^3+^ states, respectively (Figure 2d,e). This indicates that the redox state of manganese remained stable during functionalization. The survey spectrum also confirmed the presence of nitrogen and phosphorus derived from the membrane. X‐ray diffraction (XRD) analysis demonstrated that all diffraction peaks of MD@NM nanoscavengers matched well with the hausmannite Mn_3_O_4_ crystal structure (JCPDS No. 24‐0734), with no structural changes observed following surface modification (Figure 2f). FTIR spectroscopy further validated the integration of Mn_3_O_4_, DNase‐1, and membrane components. As shown in Figure 2g, MD@NM nanoscavengers exhibited characteristic absorption peaks corresponding to Mn_3_O_4_ and DNase‐1, as well as newly emerged amide (amide I/II) and phospholipid‐related bands, confirming the co‐existence of protein and membrane materials.

To assess the enzymatic activity and confirm the functional integrity of DNase‐1 post‐conjugation, we conducted agarose gel electrophoresis using exogenous DNA as substrate. As shown in Figure 2i, MD@NM nanoscavengers efficiently degraded DNA under physiological conditions, exhibiting comparable activity to free DNase‐1. This indicates that the enzymatic function of DNase‐1 was preserved during the immobilization process. Sodium dodecyl sulfate‐polyacrylamide gel electrophoresis (SDS‐PAGE) was employed to evaluate membrane protein retention on MD@NM nanoscavengers. To further evaluate the stability of MD@NM nanoscavengers under physiological conditions, DLS and zeta potential measurements were conducted after storage in PBS or 10% fetal bovine serum (FBS). The results showed that MD@NM displayed minimal variation in hydrodynamic size and surface charge in both media, suggesting excellent colloidal stability and effective resistance against serum protein adsorption‐induced aggregation. These findings demonstrate that the neutrophil membrane coating endows MD@NM with enhanced stability in complex biological environments (Figures S4 and S5). Quantitative analysis demonstrated negligible Mn^2+^ leakage under physiological conditions, supporting the high structural stability of the Mn_3_O_4_ core (Figure S6). As shown in Figure 2j, the protein banding pattern of MD@NM closely resembled that of native neutrophil membranes, indicating successful membrane coating. To further verify the preservation of inflammation‐relevant proteins, Western blotting was performed to detect chemotaxis and adhesion‐associated markers. To further evaluate the long‐term stability of DNase‐1 after encapsulation, MD@NM was incubated under PBS and oxidative conditions (H_2_O_2_) for up to 24 h, and the remaining dsDNA content was quantified. Under PBS conditions, dsDNA levels remained low and increased only slightly over time, indicating that DNase‐1 retained stability without causing excessive DNA degradation under non‐inflammatory conditions. In contrast, H_2_O_2_ treatment led to a time‐dependent increase in dsDNA levels, which is attributable to continuous ROS‐induced DNA damage and release. Notably, DNase‐1 encapsulated in MD@NM remained functional under oxidative stress, rather than undergoing abrupt inactivation, suggesting that the nanoplatform provides partial protection to DNase‐I in inflammatory microenvironments (Figure S8). As shown in Figure 2k, key proteins such as CXCR2 and LFA‐1 were well retained in MD@NM nanoscavengers, consistent with the membrane‐derived origin and essential for inflammation targeting. ROS such as superoxide anions (·O_2_^−^), hydrogen peroxide (H_2_O_2_), and hydroxyl radicals (·OH) play vital roles in cellular signaling when present at physiological levels [52, 53]. However, excessive ROS can damage cellular macromolecules, including lipids, nucleic acids, and proteins, ultimately leading to apoptosis or necrosis [54]. Thus, modulating ROS levels is essential for maintaining redox homeostasis. In terms of enzymatic mimicry, MD@NM nanoscavengers exhibited multiple ROS‐scavenging activities. Superoxide dismutase (SOD)‐like activity was confirmed using a xanthine–xanthine oxidase system, followed by WST‐8 detection. To determine an appropriate working concentration for in vitro studies, the cytocompatibility and functional efficacy of MD@NM were systematically evaluated. CCK‐8 assays revealed that MD@NM exhibited negligible cytotoxicity at concentrations up to 10 µg/mL, whereas higher concentrations showed no additional biological benefit. Based on these results, 10 µg/mL was selected as a safe and biocompatible dose. In parallel, antioxidant and ROS‐scavenging activities of MD@NM were assessed using SOD, CAT, and MB assays. The results demonstrated that MD@NM at 10 µg/mL achieved robust enzymatic activity and ROS elimination efficiency comparable to higher doses. Therefore, 10 µg/mL was chosen as an optimal concentration that balances cytocompatibility and functional performance for in vitro experiments (Figures S9–S11). As shown in Figure 2l, the SOD‐like activity increased significantly from 19.04% (bare Mn_3_O_4_) to 61.50% (MD@NM nanoscavengers). H_2_O_2_‐scavenging activity, indicative of catalase (CAT)‐like function, also showed a notable enhancement from 14.91% to 64.47% after MD@NM nanoscavengers functionalization (Figure 2m). Upon MD@NM nanoscavengers addition to H_2_O_2_ solution, the rapid generation of oxygen bubbles was observed. Dissolved oxygen monitoring further confirmed increased oxygen release over time (Figure 2n), demonstrating the potent CAT‐mimetic activity of MD@NM nanoscavengers. In addition to its SOD and CAT‐like functions, MD@NM nanoscavengers were also capable of neutralizing hydroxyl radicals (·OH), with a scavenging efficiency exceeding 75% (Figure 2o). Taken together, these results demonstrate that MD@NM nanoscavengers are structurally stable, catalytically active, and biologically functional capable of dual targeting NETs and ROS, laying a solid foundation for its in vivo therapeutic evaluation.

### 2.3 Neutrophil‐Mimetic MD@NM Nanoscavengers Eliminate ROS to Relieve Oxidative Stress In Vitro

**FIGURE 3:**
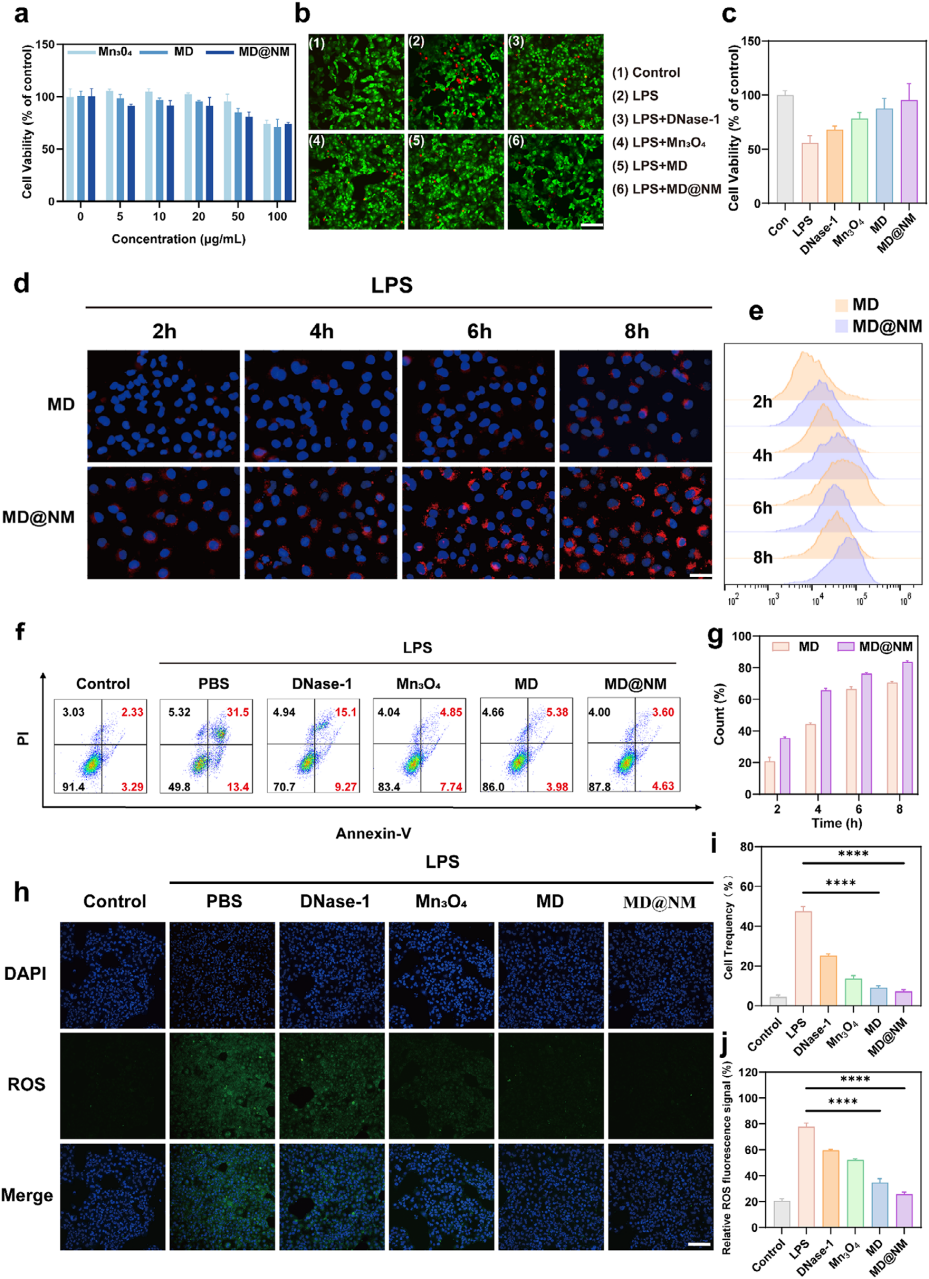
Protective effects of MD@NM nanoscavengers against oxidative damage in vitro: (a) Cell viability under various concentrations of MD@NM treatment. (b) Representative live/dead staining images using Calcein‐AM and propidium iodide (PI) to assess cell viability across treatment groups. Scale bar: 100 µm. (c) Quantification of cell survival following MD@NM treatment under oxidative stress in a dose‐dependent manner. (d) Fluorescence images of phagocytosis in LPS‐treated cells following treatment with MD or MD@NM at 2, 4, 6, and 8 h. Scale bar: 100 µm (e) Flow cytometry plots and corresponding statistical analysis (g, n = 3) of phagocytic activity across treatment groups. (f) Flow cytometric analysis of apoptosis (Annexin V/PI staining) and its quantification (i, n = 3) in different groups. (h) Representative fluorescence images of intracellular ROS levels detected by DCFH‐DA probe, and corresponding statistical analysis (j, n = 3). Scale bar: 200 µm. The data are presented as the means ± SD (n = 3). Statistical significance was determined by ANOVA. ^*^ *p* < 0.05, ^**^ *p* < 0.01, ^***^ *p* < 0.001; ns, not significant.

Failure of antioxidant defense systems leads to the accumulation of excessive ROS, resulting in cell death and inflammatory cascades [55, 56]. In view of the potent antioxidative capability of MD@NM nanoscavengers, we systematically evaluated their cytoprotective and anti‐inflammatory potential through an in vitro oxidative damage model. Prior to mechanistic studies, the cytotoxicity of MD@NM nanoscavengers was first assessed using the Cell Counting Kit‐8 (CCK‐8) assay. As anticipated, HK‐2 cells (human proximal tubular epithelial cells) and macrophages exhibited over 90% viability when incubated with various concentrations of MD@NM nanoscavengers (Figure S12), indicating negligible cytotoxicity. Based on these results, a concentration of 10 µg/mL was selected for further experimentation. At this dose, MD@NM demonstrated superior protective efficacy compared to the Mn_3_O_4_‐only group and the MD group, highlighting the synergistic advantage of membrane‐coated composite nanoscavengers (Figure 3a). To determine whether MD@NM nanoscavengers could mitigate LPS‐induced oxidative injury, Calcein‐AM/PI dual staining was employed. As shown in Figure 3b, no appreciable cell death was observed in the MD@NM group, whereas LPS treatment induced extensive cell death. Compared with free DNase‐1, Mn_3_O_4_ and uncoated MD, MD@NM markedly reduced LPS‐induced cell death, indicating that nanoparticle functionalization and neutrophil‐membrane coating are critical for cytoprotection. Moreover, we evaluated the viability of HK‐2 cells under oxidative stress. Upon LPS stimulation, cell viability decreased markedly to 55.77%, indicating severe ROS‐mediated injury. Treatment with free DNase‐1 partially alleviated the damage, increasing viability to 67.93% (a relative improvement of 1.2‐fold compared with LPS alone). Mn_3_O_4_ nanozymes provided stronger antioxidant protection, raising viability to 78.30% (1.4‐fold versus LPS). When DNase‐1 was integrated with Mn_3_O_4_ to form MD, cell viability further increased to 87.43%, reflecting synergistic DNA degradation and ROS‐scavenging functions. Notably, MD@NM nanoscavengers achieved the most pronounced protection, restoring viability to 95.26% (1.7‐fold relative to LPS and 1.1‐fold higher than MD), further underscoring the robust ROS‐scavenging and cytoprotective capacity of the nanoscavengers. (Figure 3c). To evaluate cellular uptake dynamics, confocal laser scanning microscopy (CLSM) was used to track nanoparticle internalization in a time‐dependent manner after LPS stimulation. To clarify whether the enhanced renal accumulation of MD@NM arises from active neutrophil membrane‐mediated targeting rather than altered circulation properties, functional blocking experiments were performed. MD@NM was preincubated with blocking antibodies against CXCR2 or LFA‐1, two key chemotactic and adhesion molecules expressed on neutrophil membranes, prior to cellular uptake studies. Fluorescence imaging revealed that blocking either CXCR2 or LFA‐1 significantly reduced the uptake of MD@NM at 0.5, 2, and 6 h. These results demonstrate that the targeting capability of MD@NM is critically dependent on neutrophil membrane‐derived adhesion and chemotactic molecules, thereby confirming true active targeting behavior (Figure S13). As shown in Figure 3d, MD@NM nanoscavengers displayed significantly stronger fluorescence signals at 2, 4, 6, and 8 h post‐treatment compared to MD, indicating enhanced cellular internalization, likely due to the biointerfacing properties of the neutrophil membrane. This trend was confirmed via flow cytometry, the MD group showed a progressive increase in cellular internalization, with uptake percentages of 20.87%, 44.37%, 66.43%, and 70.60% at 2, 4, 6, and 8 h, respectively. In contrast, the MD@NM group exhibited consistently higher uptake, reaching 35.43%, 65.77%, 76.27%, and 83.70% at the same time points. At the early 2 h time point, the uptake of MD@NM was already 1.7‐fold greater than MD. This advantage persisted throughout the observation window, with MD@NM surpassing MD by 21.4% at 4 h, 9.8% at 6 h, and 13.1% at 8 h. These results demonstrate that neutrophil membrane camouflage markedly enhances cellular recognition and internalization efficiency, enabling more rapid and sustained accumulation compared with uncoated MD nanoparticles. (Figure 3e,g). These findings suggest that the membrane coating contributes to both enhanced recognition and uptake of the particles by inflamed epithelial cells. Next, we investigated the anti‐apoptotic potential of MD@NM nanoscavengers using Annexin V‐FITC/PI dual staining and flow cytometry. As illustrated in Figure 3f, LPS stimulation markedly increased apoptosis to 47.50%, compared with negligible levels in untreated controls, indicating severe cell injury. Treatment with free DNase‐1 reduced apoptosis to 25.21%, representing a 47% reduction relative to LPS. Mn_3_O_4_ nanozymes conferred stronger protection, lowering apoptosis further to 13.66% (71% reduction versus LPS). When DNase‐1 was integrated with Mn_3_O_4_ (MD group), the apoptotic rate declined to 9.09%, corresponding to an 80% reduction. Remarkably, MD@NM nanoscavengers provided the most robust protection, decreasing apoptosis to only 7.30%, which is 6.5‐fold lower than LPS alone and 1.2‐fold lower than MD. Collectively, these results demonstrate that DNase‐1, Mn_3_O_4_, and neutrophil membrane coating each contribute distinct protective effects, with MD@NM achieving the most effective suppression of apoptosis in LPS‐injured HK‐2 cells. Given the established link between intracellular ROS accumulation and apoptosis, we further explored MD@NM's ROS scavenging efficiency using the DCFH‐DA probe. DCFH‐DA reacts with intracellular ROS and converts into highly fluorescent DCF, which serves as a direct indicator of ROS levels. As shown in Figure 3h, intense green DCF fluorescence was observed in the LPS group, whereas fluorescence intensity progressively declined with nanoparticle refinement, reaching the lowest level in the MD@NM group. Quantitative analysis corroborated these results (Figure 3i), showing a clear reduction in ROS levels aligned with increasing nanoparticle concentration, a finding corroborated by flow cytometry (Figure S14). Collectively, these results demonstrate that MD@NM nanoscavengers not only effectively neutralize intracellular ROS but also protect renal epithelial cells from oxidative injury, reduce inflammation‐induced apoptosis, and enhance cellular phagocytic capacity. This establishes MD@NM nanoscavengers as promising candidates for the treatment of oxidative stress‐related renal injuries, particularly in the context of SAKI.

### 2.4 Inhibitory Effects of MD@NM Nanoscavengers on NETs Formation

**FIGURE 4:**
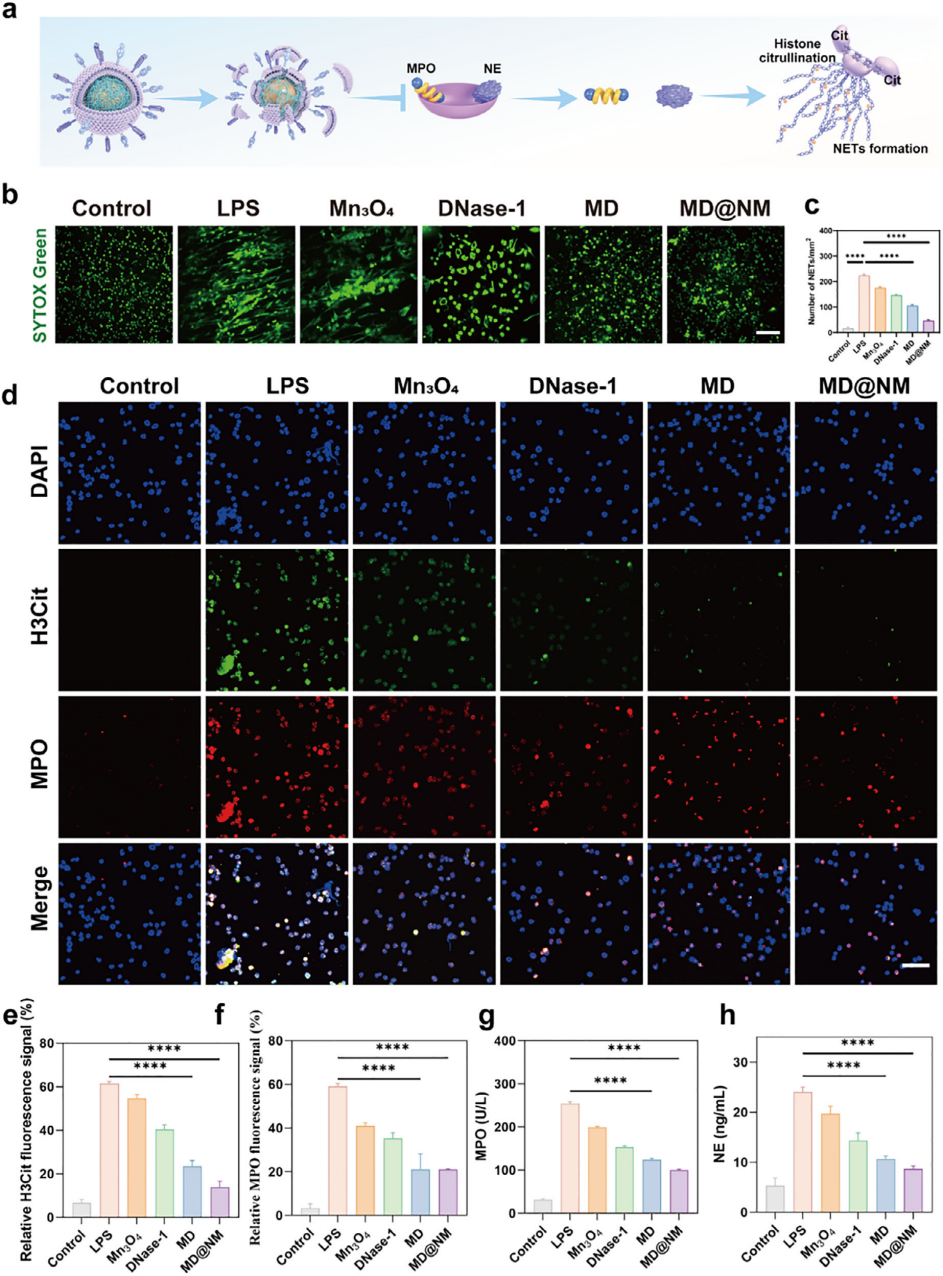
Inhibition of NETs formation by MD@NM nanoscavengers: (a) Schematic illustration of MD@NM‐mediated inhibition of NETs formation. (b) Representative confocal laser scanning microscopy (CLSM) images of neutrophil extracellular DNA (SYTOX Green) with or without LPS stimulation, followed by treatment with different formulations. Scale bar: 100 µm (c) Quantification of NETs formation based on SYTOX Green fluorescence intensity (n = 3). (d) Representative immunofluorescence images of LPS‐induced NETs stained for citrullinated histone H3 (H3Cit, green) and myeloperoxidase (MPO, red); nuclei were counterstained with DAPI (blue). (e) Quantification of relative fluorescence intensity of H3Cit (n = 3). Scale bar: 100 µm (f) Quantification of relative fluorescence intensity of MPO (n = 3). (g, h) ELISA analysis of MPO‐DNA complexes and NE levels in each treatment group (n = 3). Data are shown as the mean values ± SD (n = 3). Statistical significance was determined by ANOVA. ^*^ *p* < 0.05, ^**^ *p* < 0.01, ^***^ *p* < 0.001; ns, not significant.

Following SAKI, a robust inflammatory response triggers the rapid recruitment of neutrophils. These neutrophils can release NETs, which are web‐like structures composed of DNA, histones (particularly citrullinated histone H3, H3Cit), and granule proteins such as MPO and NE [57]. While initially protective, excessive NETs formation contributes to tissue damage and disrupts immune homeostasis. Intracellular ROS are known to drive the release of granule proteins (e.g., MPO and NE) and promote histone H3 citrullination, all of which are essential for NET formation (Figure 4a) [58]. To evaluate the capacity of MD@NM nanoscavengers to inhibit NETs release, we utilized the DNA‐binding fluorescent dye SYTOX to visualize extracellular DNA fibers. Consistent with our hypothesis, quantitative analysis revealed that SYTOX fluorescence intensity was markedly elevated following LPS stimulation, indicating extensive extracellular DNA release. Treatment with free DNase‐1 reduced the signal by approximately 22% compared with LPS, while Mn_3_O_4_ nanozymes further decreased it by about 35%. When DNase‐1 was combined with Mn_3_O_4_ to form MD, the reduction reached nearly 53%. Notably, MD@NM nanoscavengers exerted the most pronounced effect, showing a 4.8‐fold decrease relative to LPS and a 2.3‐fold reduction compared with MD. (Figure 4b,c). In addition, LPS stimulation markedly increased the expression of canonical NETs markers MPO and Cit H3 compared with the unstimulated control. Treatment with free DNase‐1 led to a moderate reduction in MPO by 31% and Cit H3 by 11%, while Mn_3_O_4_ nanozymes produced more substantial decreases of 40% and 34%, respectively. When combined, MD further suppressed MPO and Cit H3 by 64% and 62% relative to LPS, underscoring the synergistic effect of dual components. Strikingly, MD@NM exhibited the most potent inhibition, reducing Cit H3 expression by nearly 80%, approximately 4.4‐fold lower than LPS and nearly two‐fold lower than MD, thereby demonstrating its superior capacity to attenuate NETs‐associated protein expression. (Figure 4d,f). However, MD@NM nanoscavengers treatment significantly suppressed the fluorescence intensity of both markers, indicating effective inhibition of NETs formation. To further validate this observation, we measured extracellular levels of MPO and NE using ELISA. As shown in Figure 4g,h, MD@NM treatment dramatically reduced the levels of both NETs‐associated enzymes, consistent with the observed reductions in H3Cit and SYTOX staining. These results demonstrate that MD@NM nanoscavengers effectively suppress NETs formation by modulating both ROS and key molecular processes involved in chromatin decondensation and granule protein release. Together, these findings underscore the potential of MD@NM nanoscavengers to mitigate excessive NETs production, thereby alleviating NETs‐mediated inflammation and tissue injury in SAKI.

### 2.5 MD@NM Nanoscavengers Immunomodulate the cGAS‐STING Pathway and Reprogram Macrophage Polarization

**FIGURE 5:**
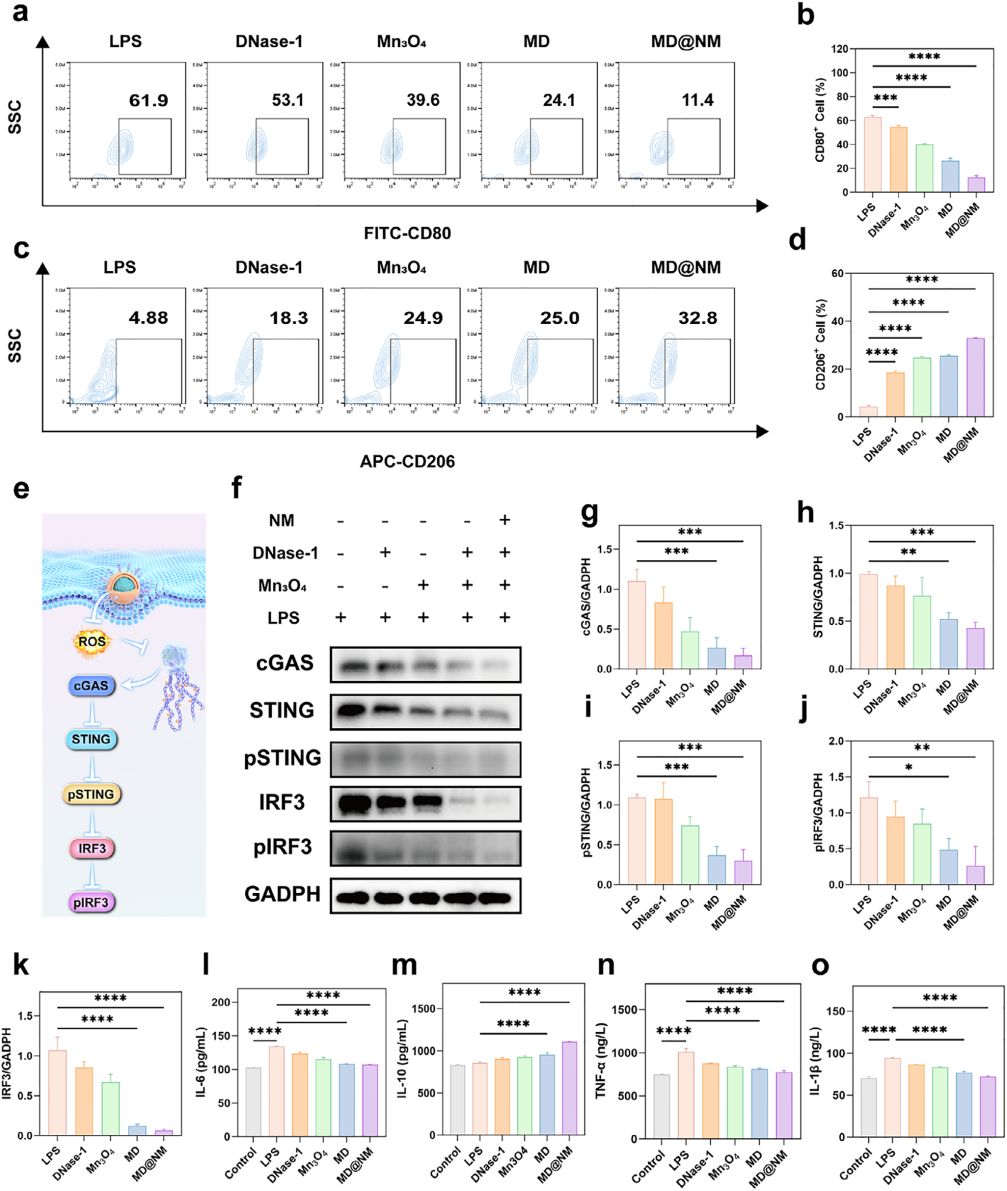
Immunomodulatory effect of MD@NM nanoscavenger on macrophages: (a) Representative flow cytometry plots showing M1‐polarized macrophages labeled with CD80 after treatment with various formulations. (b) Quantification of the proportion of CD80 M1 macrophages (n = 3). (c) Representative flow cytometry plots of M2‐polarized macrophages labeled with CD206. (d) Quantification of the percentage of CD206^+^ M2 macrophages (n = 3). (e) Schematic illustration of the proposed mechanism by which MD@NM inhibits the cGAS‐STING signaling pathway. (f) Western blot analysis and densitometric quantification of cGAS, STING, phosphorylated STING (p‐STING), IRF3, and phosphorylated IRF3 (p‐IRF3) in macrophages under different treatment conditions (n = 3). (k‐o) ELISA‐based quantification of pro‐ and anti‐inflammatory cytokines, including IL‐10, TNF‐α, IL‐6, and IL‐1β, in RAW264.7 macrophages following treatment with the indicated formulations (n = 3). Data are shown as the mean values ± SD (n = 3). Statistical significance was determined by ANOVA. ^*^ *p* < 0.05, ^**^ *p* < 0.01, ^***^ *p* < 0.001; ns, not significant.

**FIGURE 6:**
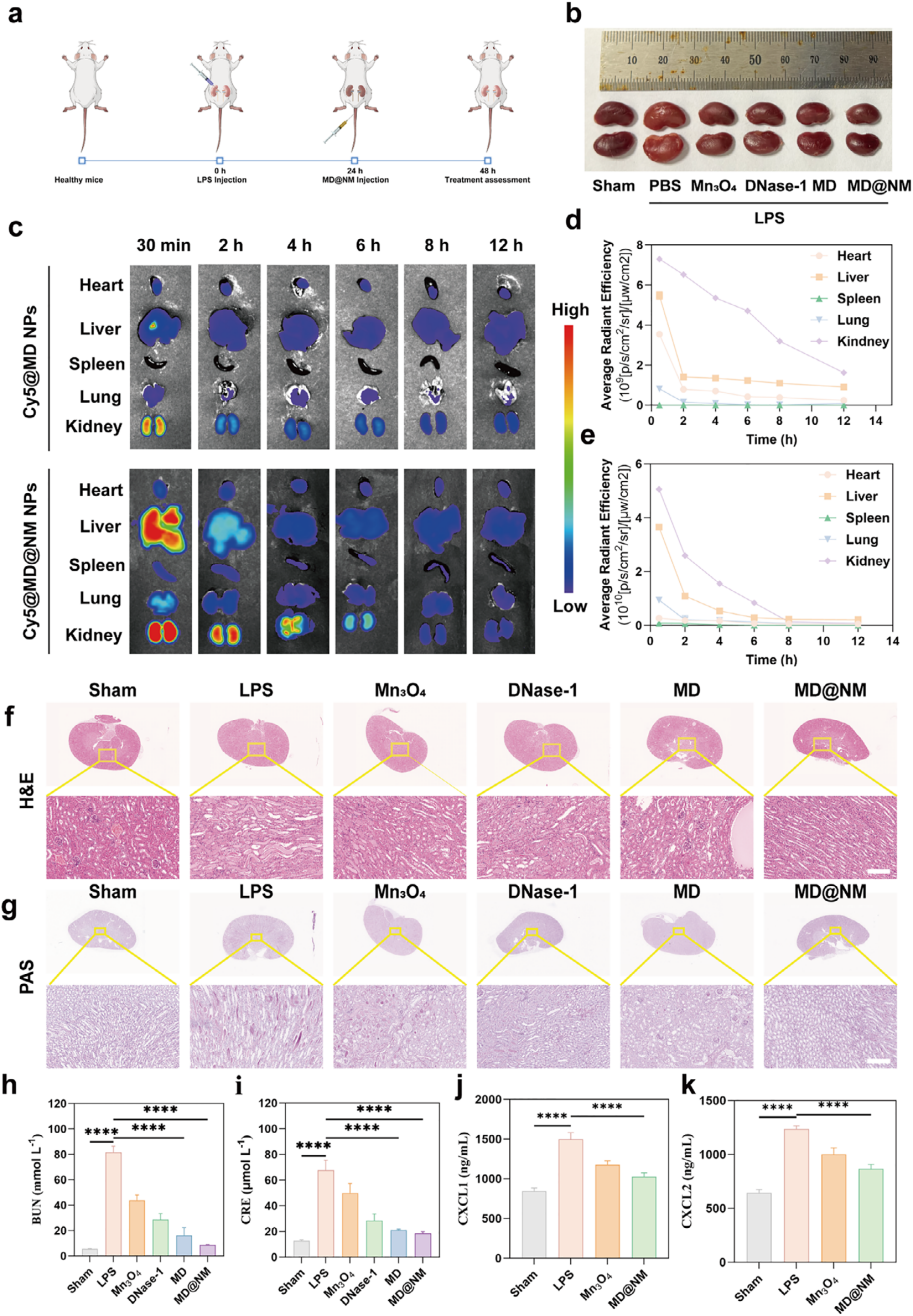
Therapeutic efficacy of MD@NM nanoscavengers in SAKI mice: (a) Schematic illustration depicting the experimental timeline for the establishment of SAKI and the subsequent treatment regimen in mice. (b) Representative images of kidneys harvested from mice in different experimental groups. (c) Ex vivo fluorescence imaging showing the biodistribution of Cy5‐labeled MD@NM nanoscavengers in major organs (heart, liver, spleen, lung, and kidney) of sham and SAKI mice at various time points post‐injection. (d‐e) Quantitative analysis of the mean fluorescence intensity in each organ based on the ex vivo imaging data. Data are presented as mean ± standard error of the mean (s.e.m.; n = 3). (f) Schematic diagram illustrating the proposed mechanism by which MD@NM inhibits the cGAS‐STING signaling pathway. Scale bar: 50 µm (g) H&E staining of kidney tissues from each group to assess histopathological alterations. Scale bar: 50 µm (h) PAS staining of renal tissues to evaluate tubular injury and glycogen deposition. (i) Quantitative measurements of serum BUN and CRE levels in SAKI mice 24 h after different treatments (n = 3). (j‐k) Expression levels of CXC chemokine ligands CXCL1 and CXCL2 were determined to assess the inflammatory microenvironment in the kidney post‐treatment. Data are shown as the mean values ± SD (n = 3). Statistical significance was determined by ANOVA. ^*^ *p* < 0.05, ^**^ *p* < 0.01, ^***^ *p* < 0.001; ns, not significant.

To further elucidate the downstream immunomodulatory effects of the MD@NM nanoscavengers, we investigated their impact on the cGAS‐STING signaling axis and macrophage polarization. As shown in Figure 5a, LPS stimulation markedly promoted the polarization of RAW264.7 macrophages toward a proinflammatory M1 phenotype, characterized by increased expression of the surface marker CD80. However, treatment with MD@NM nanoscavengers significantly reduced the proportion of CD80 cells, surpassing the effects observed in the Mn_3_O_4_ and MD groups. Quantitative analysis revealed that MD@NM nanoscavengers reduced CD80^+^ expression by approximately 50.5% (Figure 5b). Concurrently, the proportion of CD206^+^ M2‐type macrophages increased by 27.92% in the MD@NM nanoscavengers group (Figure 5c), a trend that was statistically validated by flow cytometry (Figure 5d). We hypothesized that this polarization shift was driven by the attenuation of cytosolic DNA sensing signals, particularly the cGAS‐STING pathway, as a result of NETs degradation. cGAS, a cytosolic DNA sensor, recognizes double‐stranded DNA (dsDNA) released from NETs and activates STING, which in turn triggers downstream phosphorylation of IRF3 and subsequent proinflammatory cytokine production [59]. By delivering DNase‐1, MD@NM effectively degraded extracellular DNA derived from NETs, thereby reducing the accumulation of cytosolic dsDNA and limiting cGAS activation (Figure 5e). Western blot analysis was conducted to evaluate the expression of key proteins in the cGAS‐STING axis. As shown in Figure 6f, LPS stimulation markedly activated the cGAS‐STING pathway, with cGAS, STING, p‐STING, IRF3, and p‐IRF3 all showing pronounced upregulation compared with the control. Free DNase‐1 conferred only modest reductions, lowering cGAS by 25% and p‐IRF3 by 23% relative to LPS. Mn_3_O_4_ nanozymes exerted stronger effects, decreasing cGAS, STING, and p‐STING levels by 57%, 23%, and 32%, respectively. Notably, the MD formulation further potentiated pathway inhibition, reducing cGAS and IRF3 expression by 75% and 89%, respectively. Strikingly, MD@NM exhibited the most pronounced suppression, lowering cGAS expression by 85% compared with LPS, while reducing STING and p‐STING by 57% and 72%, respectively. Moreover, IRF3 and p‐IRF3 levels were diminished by 94% and 79%, nearly 18‐fold and 5‐fold lower than LPS, respectively, indicating that the intact MD@NM nanoscavengers system possesses potent inhibitory activity against this pathway (Figure 5f–k). To determine whether the reduction in total cGAS, STING, and IRF3 protein levels was attributable to transcriptional regulation, qPCR analysis was performed to quantify their corresponding mRNA expression. The results revealed a significant downregulation of cGAS, STING, and IRF3 transcripts, which was consistent with the observed decrease in total protein levels. In parallel, Western blot analyses of pSTING and pIRF3 were repeated under optimized conditions, yielding clearer phosphorylation signals. These results further confirm that MD@NM suppresses cGAS‐STING‐IRF3 signaling at both transcriptional and post‐translational levels (Figures S15–S17). To further determine whether the suppression of cGAS‐STING signaling translated into reduced inflammatory responses, we assessed the levels of major cytokines secreted by macrophages using ELISA. As shown in Figure 5l‐o, LPS stimulation markedly enhanced pro‐inflammatory cytokines, with IL‐1β, IL‐6, and TNF‐α increasing by more than 3‐5 fold compared to baseline. Free DNase‐1 produced only a modest reduction of 8‐13%, while Mn_3_O_4_ achieved a slightly greater decrease of 12‐17%. The MD formulation further suppressed cytokine release by 19‐20%. Strikingly, MD@NM achieved the most pronounced inhibition, reducing IL‐1β, IL‐6, and TNF‐α levels by approximately 20‐25% relative to LPS, restoring them close to baseline. In contrast, the anti‐inflammatory cytokine IL‐10 was progressively elevated across groups, with MD@NM inducing a 30% increase compared to LPS, the highest among all conditions. These findings strongly support the immunomodulatory capability of MD@NM nanoscavengers in promoting M2 macrophage polarization and mitigating inflammation via inhibition of the cGAS‐STING axis.

### 2.6 In Vivo Therapeutic Efficacy of MD@NM Nanoscavengers in a Mouse Model of SAKI

Building upon the potent antioxidant performance observed in vitro, we next evaluated the in vivo therapeutic efficacy of MD@NM nanoscavengers in a murine model of SAKI. SAKI is a prevalent clinical complication characterized by high morbidity and mortality, yet current therapeutic options remain limited. Oxidative stress plays a central role in both the initiation and progression of SAKI, thereby highlighting the promise of antioxidant‐based therapies [60, 61]. To assess the therapeutic potential of MD@NM nanoscavengers in vivo, we established a standardized 24‐h LPS‐induced SAKI model in mice. MD@NM were administered intravenously (i. v.) via tail vein injection (Figure 6a). As shown in representative gross kidney images (Figure 6b), kidneys from the sham group appeared dark red and healthy, whereas those from the untreated SAKI group exhibited visible swelling and congestion, indicative of severe renal injury. Remarkably, MD@NM‐treated SAKI mice showed significant improvement in renal appearance, with kidneys resembling those of the healthy group in both color and morphology. To further investigate the biodistribution of MD@NM, we intravenously injected Cy5‐labeled MD@NM (Cy5@MD@NM NPs) into SAKI mice and harvested major organs at different time points for ex vivo fluorescence imaging (Figure 6c–e). In sharp contrast, MD@NM exhibited markedly higher renal accumulation at each time point. At 30 min, the renal signal of MD@NM was 7‐fold stronger than that of MD, and it remained 4‐fold and 3‐fold higher at 2 h and 4 h, respectively. This inflammation‐targeted distribution was likely driven by the neutrophil membrane coating, which facilitated active homing to damaged renal tissue. In contrast, the fluorescence intensity in the liver and lungs was relatively lower in the MD group, which may reflect variations in vascular permeability associated with SAKI [62]. During sepsis, increased hepatic and pulmonary permeability, together with disruption of the alveolar‐capillary barrier [62], may contribute to the enhanced accumulation of NPs in these organs. Importantly, MD@NM nanoscavengers showed prolonged renal retention and gradual signal clearance over time, suggesting both effective localization and favorable biodegradability, thereby supporting their therapeutic efficiency while minimizing systemic toxicity. Histological analysis revealed that MD@NM nanoscavengers treatment significantly mitigated LPS‐induced renal damage. Hematoxylin and eosin (H&E) staining demonstrated extensive tubular injury, swelling, and cast formation in the SAKI group, which were markedly alleviated following MD@NM nanoscavengers administration (Figure 6f). Periodic acid‐Schiff (PAS) staining further confirmed the restoration of brush border integrity in renal tubules post‐treatment (Figure 6g). Physiologically, SAKI mice exhibited markedly elevated blood urea nitrogen (BUN) and serum creatinine (CRE) levels, reflecting impaired glomerular filtration. LPS stimulation caused marked increases in both indicators relative to controls, confirming severe renal dysfunction. Free DNase‐1 led to only a modest reduction, decreasing CRE and BUN by less than 1.4‐ to 2‐fold compared with LPS. Mn_3_O_4_ nanozymes produced a stronger effect, lowering both parameters by approximately 2.4‐ to 3.3‐fold. The MD formulation further improved efficacy, reducing CRE and BUN by nearly 3.2‐ to 6.6‐fold relative to LPS. Strikingly, MD@NM exhibited the most potent activity, suppressing CRE and BUN by 3.7‐ and 9.5‐fold compared with LPS, restoring both indices close to baseline. These findings demonstrate a stepwise enhancement in renoprotective efficacy across formulations, with MD@NM providing the most substantial recovery of kidney function within 24 h. (Figure 6h,i). Considering the critical role of neutrophil infiltration and NETs formation in SAKI pathogenesis, we next quantified renal levels of proinflammatory chemokines. ELISA analysis revealed significant upregulation of CXCL1 and CXCL2 in the kidneys of SAKI mice, which was significantly suppressed by MD@NM nanoscavengers treatment (Figure 6j,k). This is consistent with the observed inhibition of NETs formation and supports the immunomodulatory function of MD@NM nanoscavengers in ameliorating renal inflammation.

### 2.7 Immunotherapeutic Effects of MD@NM Nanoscavengers in SAKI Mice

**FIGURE 7:**
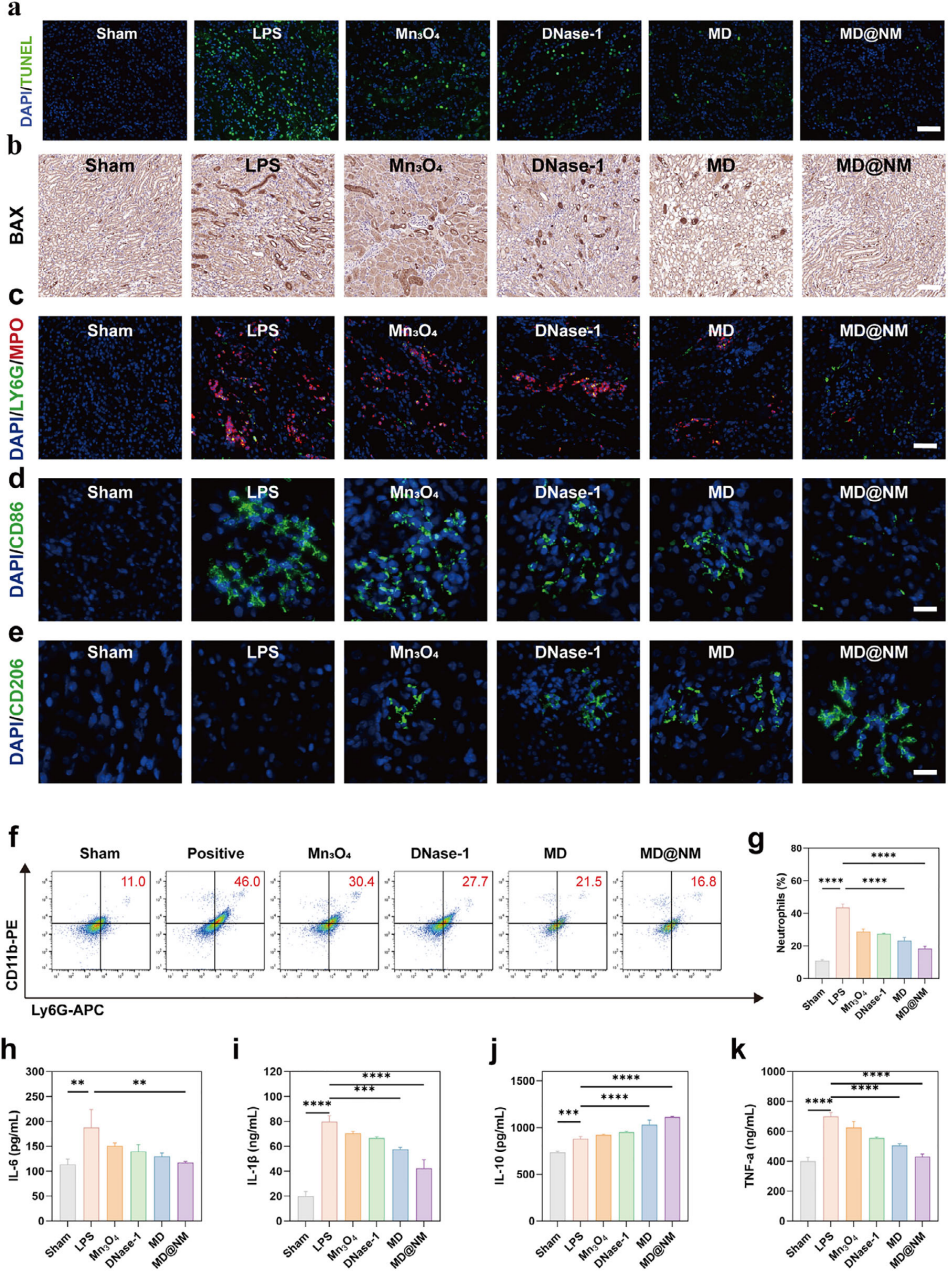
Therapeutic Effects of MD@NM on Renal Inflammation and Cell Death in SAKI Mice: (a) TUNEL staining of kidney tissues from different treatment groups to assess apoptotic cell death. Scale bar: 50 µm (b) Immunohistochemical analysis of pro‐apoptotic protein BAX in renal sections following various treatments. Scale bar: 200 µm (c) Representative immunofluorescence images showing co‐localization of Ly6G (green, neutrophils) and H3Cit (red, a marker of neutrophil extracellular traps, NETs), with nuclei counterstained by DAPI (blue). Scale bar: 50 µm (d‐e) Representative immunofluorescence images of macrophage polarization markers: CD86 (green, M1 phenotype) and CD206 (green, M2 phenotype) in kidney tissues of each group. Scale bar: 200 µm (f) Flow cytometry plots of neutrophils isolated from renal tissues, with (g) corresponding quantitative analysis. (h‐k) ELISA‐based quantification of pro‐ and anti‐inflammatory cytokines including IL‐10, TNF‐α, IL‐6, and IL‐1β in kidney homogenates from healthy and SAKI mice after different treatments (n = 3). Data are shown as the mean values ± SD (n = 3). Statistical significance was determined by ANOVA. ^*^ *p* < 0.05, ^**^ *p* < 0.01, ^***^ *p* < 0.001; ns, not significant.

To further elucidate the immunoregulatory properties of MD@NM nanoscavengers in vivo, we evaluated their effects on renal immune cell infiltration, macrophage polarization, and cytokine profiles in the LPS‐induced SAKI model. Histopathological and immunological assessments were performed to determine their role in modulating inflammation and promoting immune homeostasis. TUNEL staining demonstrated significant tubular epithelial apoptosis in the SAKI group, which was substantially reduced following MD@NM nanoscavengers treatment (Figure 7a). Compared with PBS, Mn_3_O_4_, and MD groups, MD@NM nanoscavengers markedly decreased the number of apoptotic nuclei, indicating robust anti‐apoptotic effects. Immunohistochemistry for BAX, a pro‐apoptotic protein, further confirmed reduced BAX expression in renal tissue of MD@NM‐treated mice (Figure 7b), underscoring its cytoprotective capability under septic stress. Given the pathological contribution of NETs, dual immunofluorescence staining for Ly6G (neutrophils) and H3Cit (NETs marker) was performed. The SAKI group exhibited extensive co‐localization of Ly6G and H3Cit, indicative of abundant NETs formation (Figure 7c). MD@NM nanoscavengers treatment significantly reduced both neutrophil accumulation and H3Cit deposition, demonstrating effective inhibition of in vivo NETs formation. To examine macrophage polarization, we analyzed CD86 (M1 marker) and CD206 (M2 marker) expression in renal tissues. SAKI kidneys contained elevated levels of pro‐inflammatory CD86^+^ M1 macrophages, which were markedly downregulated by MD@NM nanoscavengers (Figure 7d). Conversely, CD206^+^ anti‐inflammatory M2 macrophages were significantly enriched in the MD@NM nanoscavengers group (Figure 7e), indicating that MD@NM reprograms macrophages toward an immunoresolving phenotype. Flow cytometry corroborated these histological findings, showing a striking expansion of Ly6G neutrophils in the kidneys of SAKI mice, consistent with acute inflammatory infiltration. Administration of free DNase‐1 or Mn_3_O_4_ alone provided only partial relief, each reducing neutrophil levels by less than two‐fold compared with LPS, indicating limited efficacy in curbing cellular recruitment. In contrast, the combined MD formulation exerted a more robust effect, decreasing infiltration by nearly two‐fold. Remarkably, MD@NM achieved the strongest suppression, lowering Ly6G neutrophils by more than 2.3‐fold relative to LPS. This progressive reduction across treatment groups underscores that MD@NM exhibits the strongest efficacy in blocking neutrophil‐driven injury. (Figure 7f,g). We next evaluated systemic cytokines in serum to assess inflammatory responses. Compared with healthy controls, the SAKI model exhibited a robust elevation of TNF‐α, IL‐6, and IL‐1β. Free DNase‐1 produced only a slight attenuation, reducing cytokine levels by less than 1.2‐fold relative to LPS. Mn_3_O_4_ nanoparticles exerted stronger effects, lowering pro‐inflammatory cytokines by approximately 1.3‐fold. The MD formulation provided a further improvement, suppressing cytokines by nearly 1.5‐fold compared with LPS. Remarkably, MD@NM displayed the most potent activity, reducing TNF‐α, IL‐6, and IL‐1β by nearly 1.7‐ to 2‐fold compared with LPS, thereby restoring them toward baseline. (Figure 7i–k). In contrast, IL‐10 exhibited a progressive elevation across treatments. Relative to LPS, IL‐10 levels increased 1.05‐fold with DNase‐1, 1.1‐fold with Mn_3_O_4_, and 1.2‐fold with MD, while MD@NM achieved a pronounced 1.3‐fold increase. These results highlight a stepwise enhancement of anti‐inflammatory efficacy, with MD@NM demonstrating superior systemic immunomodulation through simultaneous suppression of pro‐inflammatory cytokines and augmentation of IL‐10 (Figure 7h), further confirming the immunomodulatory role of MD@NM nanoscavengers. In summary, MD@NM nanoscavengers exert multifaceted immunotherapeutic effects in SAKI by attenuating tubular apoptosis, suppressing NETs formation, limiting neutrophil infiltration, and rebalancing macrophage phenotypes and cytokine responses. These coordinated actions underscore their potential as a promising nano‐immunotherapeutic for sepsis‐induced AKI.

### 2.8 MD@NM Nanoscavengers Reprograms the Renal Transcriptome and Modulates the NETs‐cGAS‐STING Axis in SAKI

**FIGURE 8:**
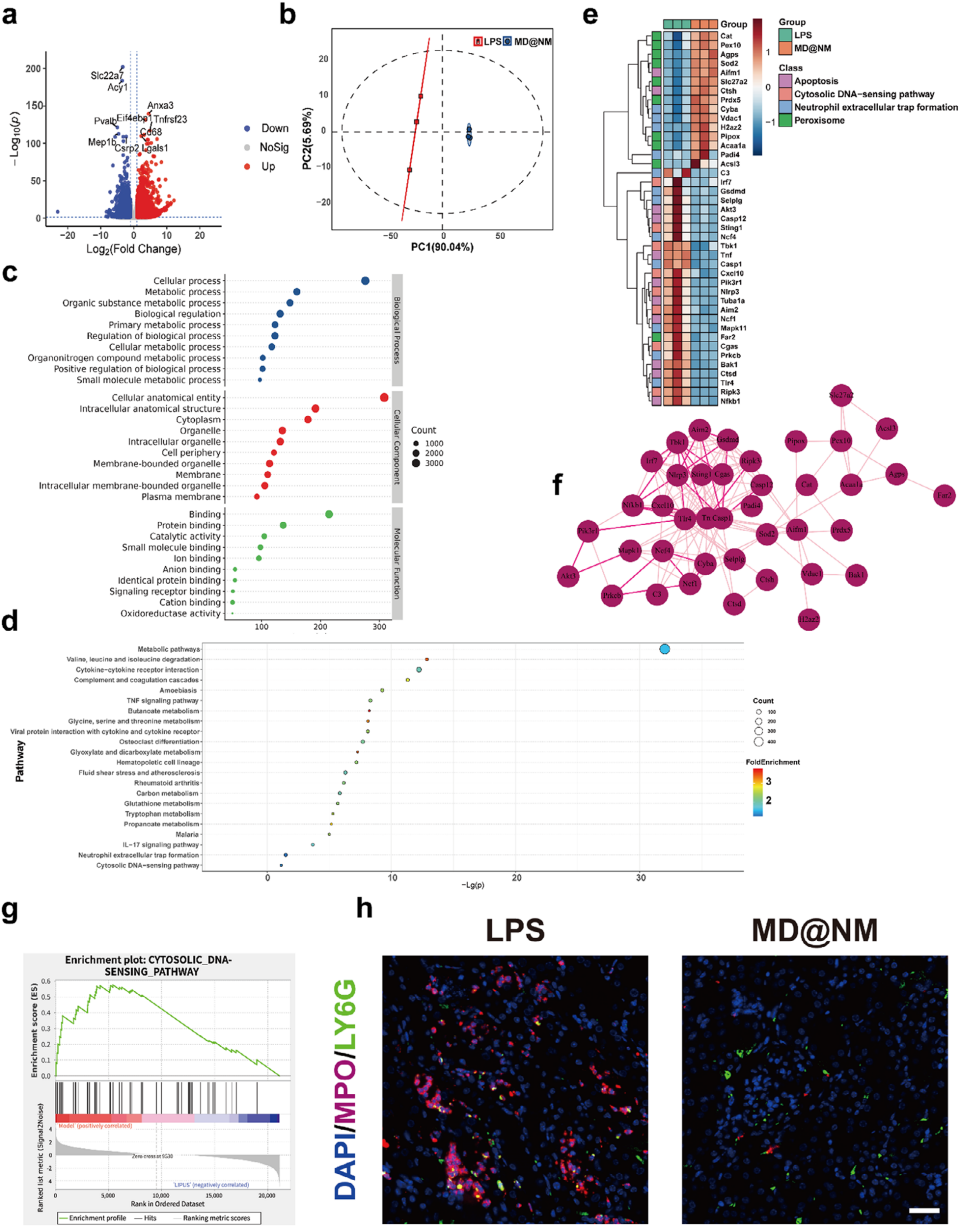
MD@NM nanoscavengers reprogram the renal transcriptome and suppress the NETs‐cGAS‐STING axis in SAKI. (a) DEGs volcano plot between sham and MD@NM groups. (b) PCA showing MD@NM transcriptional profiles shift toward sham. (c, d, g) GO and KEGG analyses indicating suppression of NET formation, cGAS‐STING signaling, cytokine production, and innate immune activation. (e) Heatmap showing downregulation of key NETs‐ and inflammation‐related genes (Elane, Mpo, Cgas, Sting1, Ifnb1, Cxcl10). (f) PPI network illustrating interconnectivity among NETs and inflammation‐associated genes. (h) Immunofluorescence showing NETs accumulation in LPS kidneys but nearly absent after MD@NM treatment (n = 3). Scale bar: 50 µm. Data are shown as the mean values ± SD (n = 3). Statistical significance was determined by ANOVA. ^*^ *p* < 0.05, ^**^ *p* < 0.01, ^***^ *p* < 0.001; ns, not significant.

To further elucidate the therapeutic mechanisms of MD@NM nanoscavengers in SAKI, we performed RNA sequencing on renal tissues from the sham and MD@NM nanoscavengers groups. This aimed to investigate the transcriptomic modulation of NETs‐related inflammation and the cGAS‐STING signaling pathway. Differential expression analysis revealed a substantial number of DEGs between the two groups, with most inflammation‐associated genes significantly downregulated following MD@NM nanoscavengers treatment (Figure 8a). PCA further demonstrated that the transcriptional profile of the MD@NM nanoscavengers group shifted closer to that of the sham group, suggesting marked attenuation of the inflammatory state (Figure 8b). GO and KEGG pathway enrichment analyses indicated that MD@NM nanoscavengers markedly inhibited biological processes and signaling pathways associated with NET formation, cGAS‐STING activation, type I interferon response, proinflammatory cytokine production, and innate immune activation (Figure 8c,d,g). Heatmap clustering further confirmed the downregulation of key genes involved in NETs formation, redox stress, apoptosis, and inflammation, such as Elane, Mpo, Cgas, Sting1, Ifnb1, and Cxcl10, following MD@NM nanoscavengers intervention (Figure 8e). To further elucidate the potential interplay among genes dysregulated in SAKI, we constructed a protein‐protein interaction (PPI) network focusing on key factors associated with NETs formation, oxidative stress, apoptosis, and inflammation. The resulting network revealed strong interconnectivity among Elane, Mpo, Cgas, Sting1, Ifnb1, and Cxcl10, suggesting that these genes may function synergistically to drive the inflammatory cascade in SAKI (Figure 8f). Transcriptomic findings were further corroborated by immunofluorescence analysis, which revealed extensive NETs accumulation in LPS group kidneys, whereas NETs were nearly absent in the MD@NM group (Figure 8h). These findings demonstrate that MD@NM nanoscavengers not only effectively scavenges NETs and reduces extracellular DNA burden but also disrupts the dsDNA‐triggered amplification of cGAS‐STING signaling. To further validate the transcriptomic findings, qPCR was performed to examine the expression of representative genes involved in the NETs‐cGAS‐STING signaling pathway. Consistent with the RNA‐seq results, renal mRNA levels of cGAS, STING, and IRF3 were significantly upregulated in SAKI mice, whereas MD@NM treatment markedly suppressed their expression. These qPCR results provide functional validation of the transcriptomic analysis and confirm that MD@NM modulates the NETs‐cGAS‐STING axis at the transcriptional level in vivo (Figure S18). To further establish a causal relationship between dsDNA clearance and suppression of cGAS‐STING signaling, rescue experiments were performed by supplementing exogenous dsDNA as a STING agonist. Western blot analysis revealed that exogenous dsDNA markedly reactivated cGAS and STING expression, whereas MD@NM treatment significantly attenuated this dsDNA‐induced reactivation. These results provide direct functional validation that dsDNA elimination is a critical upstream mechanism through which MD@NM suppresses the cGAS‐STING pathway (Figure S19). These results in a reprogramming of the renal immune transcriptome and suppression of downstream inflammatory cascades. The observed transcriptomic changes are consistent with prior histological findings of reduced NETs deposition and decreased inflammatory markers, providing robust evidence that MD@NM nanoscavengers exert its therapeutic effects in SAKI by precisely targeting the NETs‐cGAS‐STING pathway.

### 2.9 In Vivo Biosafety Assessment of MD@NM

As a developed nanomaterial for intravenous injection in the treatment of SAKI, the in vivo safety of MD@NM nanoscavengers is of paramount importance. To comprehensively evaluate the biocompatibility of MD@NM nanoscavengers, we conducted a series of hematological, biochemical, and histopathological assessments in healthy C57BL/6J mice. Blood biochemical analyses showed no significant alterations in alanine aminotransferase (ALT), aspartate aminotransferase (AST), BUN, or CRE levels across all treated groups, indicating minimal hepatotoxicity and nephrotoxicity (Figure S20). Similarly, complete blood count (CBC) tests revealed no apparent abnormalities in white blood cells, red blood cells, platelets, or hemoglobin levels (Figure S21), further supporting the hematological safety profile of MD@NM nanoscavengers. Histological examination of major organs, including the heart, liver, spleen, lung, and kidney, showed no observable tissue damage or inflammatory infiltration following treatment (Figure S22). Long‐term biosafety was further assessed by hematological analysis and histopathological examination of major organs (heart, liver, spleen, lung, and kidney) at 7, 14, and 21 days post‐injection. No noticeable pathological abnormalities or inflammatory lesions were observed, confirming the favorable long‐term biocompatibility of MD@NM in vivo (Figures S23 and S24). To further elucidate the pharmacokinetic behavior and long‐term biosafety of MD@NM in vivo, time‐dependent ICP‐MS analysis was performed to quantify Mn concentrations in blood, kidney, and liver at 0, 2, 4, 8, 12, 24, 36, and 72 h post‐injection. The blood Mn concentration exhibited a gradual decline over time, enabling estimation of the circulation half‐life of MD@NM. Notably, renal Mn levels increased at early time points and subsequently decreased, suggesting effective renal clearance without prolonged retention. Meanwhile, hepatic Mn accumulation showed a transient distribution followed by progressive elimination. These results indicate favorable pharmacokinetics and efficient systemic clearance of MD@NM, further confirming its in vivo biosafety (Figure S25). Furthermore, hemolysis assays demonstrated negligible lysis of red blood cells upon exposure to MD@NM nanoscavengers, with hemolysis rates remaining well below the 5% threshold considered safe for intravenous formulations (Figure S26). Collectively, these findings confirm the favorable biosafety and hemocompatibility of MD@NM nanoscavengers, supporting their potential for clinical translation.

## 3 Conclusion

In summary, this study presents the development of a neutrophil membrane‐coated Mn_3_O_4_/DNase‐1 (MD@NM) as the multifunctional therapeutic platform for SAKI. By integrating the intrinsic ROS‐scavenging properties of Mn_3_O_4_ with DNase‐1‐mediated degradation of extracellular DNA and inflammation‐targeted delivery via neutrophil membranes, MD@NM nanoscavengers address multiple pathological drivers of SAKI, including oxidative stress, NETs overproduction, and dysregulated immune activation. Comprehensive in vitro and in vivo assessments demonstrated that MD@NM nanoscavengers not only effectively mitigate oxidative stress and prevent tubular epithelial apoptosis but also suppress NETs formation, attenuates activation of the cGAS‐STING signaling axis, and reprogram macrophages toward an anti‐inflammatory M2 phenotype. These combined effects alleviate renal inflammation, restore cytokine balance, and improve both structural and functional recovery of the kidney in SAKI models. By targeting upstream triggers and downstream effectors of the inflammatory cascade simultaneously, MD@NM nanoscavengers offer a promising nano‐immunotherapeutic strategy for sepsis‐induced organ injury. By precisely targeting the inflammatory microenvironment and synergistically eliminating NETs and ROS while modulating the cGAS‐STING signaling pathway, this neutrophil‐mimetic nanoscavenger demonstrates significant promise as a next‐generation immunotherapeutic strategy for SAKI.

## 4 Experimental Section

### 4.1 MD@NM‐Mediated Modulation of Macrophage Polarization In Vitro

RAW 264.7 macrophages were seeded in six‐well plates and stimulated with lipopolysaccharide (LPS, 1 µg mL^−^^1^) to induce M1 polarization. After 6 h of LPS activation, culture media were replaced with conditioned supernatants derived from NETs co‐cultured with Mn_3_O_4_, free DNase‐1, MD, or MD@NM, followed by incubation for an additional 24 h. Cells were then harvested for phenotypic analysis of macrophage polarization. For immunofluorescence imaging, treated cells were fixed with 4% paraformaldehyde, permeabilized, and blocked before incubation with primary antibodies against CD80 (M1 marker) and CD206 (M2 marker) at 4 °C overnight. After washing, cells were incubated with appropriate fluorescent secondary antibodies for 1 h at room temperature, counterstained with DAPI, and visualized using confocal laser scanning microscopy (CLSM). In parallel, macrophages subjected to the same treatments were collected for flow cytometric analysis. Cells were stained with FITC‐conjugated anti‐mouse CD80 and APC‐conjugated anti‐mouse CD206 antibodies to quantify surface expression of M1 and M2 markers, respectively. Data were acquired using a flow cytometer and analyzed to determine the proportion of polarized macrophage subsets.

### 4.2 In Vivo Evaluation of the Therapeutic Efficacy of MD@NM in SAKI Mice

Male C57BL/6J mice were randomly assigned to experimental groups, and sepsis‐associated acute kidney injury (SAKI) was induced by intravenous injection of lipopolysaccharide (LPS, 10 mg kg^−^^1^) via the tail vein. Therapeutic interventions and subsequent analyses were performed 24 h after SAKI establishment. All animal procedures were approved by the Institutional Animal Care and Use Committee of the Second Affiliated Hospital of Chongqing Medical University and were conducted in strict accordance with the Regulations on the Administration of Laboratory Animals of China. Renal function was assessed by measuring serum creatinine (CRE) and blood urea nitrogen (BUN) levels. Kidney tissues were harvested for histopathological evaluation using hematoxylin and eosin (H&E) and periodic acid‐Schiff (PAS) staining. In addition, tubular cell apoptosis was examined by terminal deoxynucleotidyl transferase dUTP nick end labeling (TUNEL) immunofluorescence staining.

### 4.3 Animals experiments

C57BL/6J mice (6‐8 weeks, male) were purchased by Enswell Biotechnology Ltd (Chongqing, China). All animal experiments were approved by the Ethics Committee of Chongqing Medical University, Approval No. IACUC‐SAHCQMU‐2025‐0127.

### 4.4 Data Analysis

All the experiments were repeated at least three times. Student's unpaired or paired t‐tests were used to analyze the significance of the differences between two groups with GraphPad Prism 9.5 software (GraphPad Software, San Diego, CA). We used unpaired multiple t‐tests and analysis of variance (ANOVA) to analyze differences among multiple groups. The statistical tests were two‐sided, and values of *p* < 0.05 were considered statistically significant.

## Conflicts of Interest

The authors declare no conflicts of interest.

## Supporting information




**Supporting File**: advs74420‐sup‐0001‐SuppMat.docx.

## Data Availability

The data that support the findings of this study are available from the corresponding author upon reasonable request.
