# Supplementary material for: Neutrophil‐Mimetic Nanoscavengers Target the Inflammatory Microenvironment to Eliminate NETs/ROS and Immunomodulate cGAS‐STING Signaling in Septic AKI
Source: Adv Sci (Weinh). 2026 Feb 15;13(23):e21861. doi: 10.1002/advs.202521861 (PMC13104083; doi:10.1002/advs.202521861)
Supplement: Supplementary file 1 — Supporting File: advs74420‐sup‐0001‐SuppMat.docx. [file ADVS-13-e21861-s001.docx]

**Supplementary Information**

**Neutrophil-mimetic Nanoscavengers Target the Inflammatory Microenvironment to Eliminate NETs/ROS and Immunomodulate cGAS-STING Signaling in Septic AKI**

*Zening Z**hang, Chenxi Zhang, Ranran Luo, Qiuchi Wu, Pengchen Ren**, Xinyu Liu, Yingying Luo, Zhongsheng Xu*, Xiaojing He*, Yun Liu**

**Materials and Methods**

**Materials**

Manganese acetate (Mn (OAc)_2_·4H_2_O) was purchased from Sigma (Shanghai, China). DNase-1 (D8071) was obtained from Solarbio (Beijing, China). Minimum Essential Medium (MEM; PM150410) and Dulbecco’s Modified Eagle Medium (DMEM; PM150210) were purchased from Pricella (Wuhan, China). Paraformaldehyde (P0099), penicillin-streptomycin (C0222), trypsin (C0201), DAPI staining solution (C1002), Cell Counting Kit-8 (CCK-8; C0037), Calcein-AM/PI staining kit, and SDS-PAGE kit (P0012A) were provided by Beyotime (Shanghai, China). Sulfo-Cyanine5 (Cy5) was purchased from MedChemExpress (New Jersey, USA). Fetal bovine serum (FBS; 10100147) was supplied by Gibco (Thermo Fisher Scientific, Shanghai, China). Annexin V-FITC/PI apoptosis detection kit (E-CK-A211) was obtained from Elabscience (Wuhan, China). Calcein-AM/PI dual-staining kit (C542) and ROS assay kit (AD10) were purchased from Dojindo Laboratories (Kumamoto, Japan). ELISA kits for IL-6 (AF2163-A), TNF-α (AF2132-A), IL-10 (AF2176-A), and IL-1β (AF2040-A) were purchased from Aifang (Hunan, China). Anti-Histone H3 (AB281584) was purchased from Abcam (Cambridge, MA, USA), and Phospho-STING (PA5-105674), cGAS (703149), and Ly-6G (17-9668-80) antibodies were obtained from Thermo Fisher Scientific (Waltham, MA, USA).

**Isolation of Neutrophils from Bone Marrow**

Male C57BL/6 mice (22-25 g) were anesthetized, sacrificed via cervical dislocation, and immersed in 75% ethanol for 5 min. Hind limbs were dissected to expose and excise femurs and tibias. Bone marrow was flushed with RPMI 1640 medium, filtered through a 70 μm strainer, and centrifuged at 450 × g for 10 min. Neutrophils were subsequently isolated using a Solarbio isolation kit according to the manufacturer’s protocol.

**Extraction of Neutrophil Membranes**

Isolated neutrophils were resuspended in membrane buffer and disrupted on ice using a high-speed disperser (4200 rpm, 2 min). The lysate was centrifuged at 20,000 × g for 15 min at 4 °C to remove debris. The supernatant was then centrifuged at 100,000 × g for 35 min at 4 °C. The pellet was washed with 0.2 mM EDTA solution, followed by another ultracentrifugation (100,000 × g, 35 min, 4 °C) to collect purified neutrophil membranes (NM), which were stored at -80 °C for later use.

**Synthesis and Characterization of MD@NM**

Mn_3_O_4_ nanozymes were synthesized via a solvothermal method. Briefly, 0.408 g of manganese acetate was dissolved in 20 mL of ethanol with stirring for 20 min until fully dissolved, yielding a pink solution. The solution was transferred to a Teflon-lined stainless steel autoclave and heated at 120 °C for 24 h. After cooling, the resulting brown solution was centrifuged, and the product was washed three times with ethanol and deionized water (12,000 rpm). The Mn_3_O_4_ precipitate was resuspended in ethanol for storage. To prepare MD NPs, Mn_3_O_4_ nanozymes were centrifuged to remove ethanol and washed with Milli-Q water (3×) before dispersion in DNase-1 solution. The mixture was sonicated at 4 °C for 2 h, followed by centrifugation (12,000 rpm) and Milli-Q washes to remove unbound DNase-1. Neutrophil membranes were then mixed with the MD NPs at a membrane protein-to-core weight ratio of 2:1, followed by 2-3 min of sonication on ice. The mixture was centrifuged at 14,000 rpm for 50 min at 4 °C to remove excess membranes, yielding MD@NM NPs. Cy5 was loaded into MD@NM for in vitro and in vivo imaging. Transmission electron microscopy (TEM; HT7700, Hitachi, Japan) was used to observe the morphology of Mn_3_O_4_ and MD@NM. Hydrodynamic size and zeta potential were measured using a Zetasizer (Malvern, UK). X-ray photoelectron spectroscopy (XPS; ESCALAB 250Xi, Thermo Fisher Scientific) and Fourier-transform infrared spectroscopy (FTIR; Nicolet iS5, Thermo Fisher Scientific, USA) were used to characterize the chemical composition and confirm the presence of Mn_3_O_4_, DNase-1, and membrane components.

**Verification of Membrane-Associated Proteins**

Coomassie blue staining and Western blotting were performed as described previously to confirm the retention of neutrophil membrane proteins. Briefly, NM, intact neutrophils, Mn_3_O_4_, and MD@NM were lysed on ice in lysis buffer (Solarbio, Beijing, China) for 10 min. Lysates were centrifuged at 12,000 × g for 10 min, and supernatants were mixed with SDS loading buffer and boiled for 5 min. Equal protein amounts (20 μg) were loaded per lane for SDS-PAGE. Gels were stained with Coomassie blue (Beyotime, China) or transferred to PVDF membranes (0.22 μm, Millipore, USA) for Western blotting. After blocking with 5% nonfat milk (2 h, 37 °C), membranes were incubated overnight at 4 °C with primary antibodies, followed by secondary antibodies. Bands were visualized using BeyoECL Plus (P0018S, Beyotime).

**Measurement of •OH scavenging**

The·•OH scavenging activity was evaluated using the methylene blue (MB) discoloration method based on the Fenton reaction. Briefly, hydroxyl radicals were generated at 37 °C by reacting Fe²⁺ with H_2_O_2_. MB was employed as a probe molecule, as •OH oxidatively degraded MB, leading to a gradual decrease in its characteristic absorbance at 617 nm. In practice, FeSO_4_ and H_2_O_2_ solutions were first mixed and allowed to react for 5 min to generate·•OH. Subsequently, ultrapure water and sample solutions from different treatment groups were added and incubated for 20 min. Finally, MB solution was introduced into the reaction mixture. The colorimetric changes were recorded, and the absorbance at 617 nm was measured using a microplate reader.

**Measurement of H_2_O_2_ scavenging**

The ability of nanoscavenger to scavenge H_2_O_2_ was assessed using the H_2_O_2_ detection

kit. H_2_O_2_ reacts with ammonium molybdate to form a stable yellow complex, which exhibits an absorbance peak at 405 nm. Different nanozymes were incubated with H_2_O_2_ (2 mM) at 37 °C for 24 h. After the reaction, the concentration of remaining H_2_O_2_ was determined following the manufacturer’s instructions, and the capacity of CeLutNCs to eliminate H_2_O_2_ was calculated

**Evaluation of SOD-like activity**

The SOD-like activity of nanoscavenger was evaluated using a SOD assay kit. a superoxide anion assay kit based on the WST-1 method was applied. Superoxide anions reduce WST-1 to a water-soluble formazan dye, which exhibits strong absorbance at 450 nm. After co-incubation with nanoparticles, the suppression of absorbance indicated the ·O_2_⁻ scavenging activity. The percentage scavenging activity for each ROS species was calculated using the formula:

Scavenging Activity (%) =1$-\frac{\text{A}_{\text{sample}}}{\text{A}_{\text{control}}}\times100\%$

**Cell Culture and Cytotoxicity Assay**

HK-2 human renal tubular epithelial cells and RAW 264.7 murine macrophages (Pricella, Wuhan, China) were cultured in MEM or DMEM supplemented with 10% FBS, 100 U/mL penicillin, and 100 μg/mL streptomycin at 37 °C in a humidified 5% CO₂ incubator. Cytotoxicity of MD@NM was assessed using the CCK-8 assay. Cells were seeded at 5 × 103 cells/well in 96-well plates and incubated with different formulations for 24 h prior to viability assessment. Hemolysis assays were performed by incubating 300 μL of murine red blood cell suspension (0.9% NaCl) with nanozymes

at 37 °C for 2 h.

**Cellular Uptake and Functional Assays**

HK-2 cells (1.5 × 105/well) were seeded into 12-well plates and grown overnight on confocal dishes. Cells were treated with Cy5-labeled MD@NM with or without LPS for 6 h, followed by imaging with a Zeiss LSM 780 NLO confocal microscope to observe endocytosis. For cytoprotection studies, cells were pretreated with MD@NM for 1 h before 24 h of LPS exposure. Viability was assessed by CCK-8 at 450 nm. Annexin V-FITC/PI and Calcein-AM/PI staining were used for apoptosis and cytotoxicity analyses by confocal microscopy. ROS levels were measured using 2',7'-dichlorodihydrofluorescein diacetate (DCFH-DA; 1 μM, 30 min). Fluorescence intensity was quantified by flow cytometry and confocal microscopy. NETs formation was induced by treating neutrophils with LPS for 6 h, followed by fixation with 4% paraformaldehyde (10 min, 4 °C). Cells were stained with rabbit anti-H3Cit and anti-MPO antibodies overnight at 4 °C, followed by secondary antibodies (1 h). Nuclei were counterstained with DAPI, and images were captured by confocal microscopy. Cell supernatants were collected for cytokine ELISA per manufacturer’s instructions.

**NETs-macrophage co-culture assay.**

NETs were isolated from activated neutrophils as described above. RAW264.7 cells (or bone marrow–derived macrophages, BMDMs) were seeded in 6-well plates and incubated with NETs to establish an inflammatory co-culture system. Subsequently, cells were treated with MD@NM for the indicated time. Macrophage polarization was evaluated by analyzing the expression of M1 and M2-associated markers using immunofluorescence staining, flow cytometry, and qPCR analysis.

**Western Blot Analysis.**

Cells were lysed for protein extraction, and equal protein amounts (20 μg) were separated via 10% SDS-PAGE and transferred to PVDF membranes (0.22 μm, Millipore). Primary antibodies are detailed in the Materials section. Bands were visualized using BeyoECL Plus (Beyotime) and quantified via ImageJ (v1.8.0). All experiments were performed in triplicate.

**Biosafety and Biodistribution**

The in vivo toxicity of MD@NM was assessed in C57BL/6 mice under approval by the Ethics Committee of Chongqing Medical University (Approval No. IACUC-SAHCQMU-2024-00079; Permit No. 223, 2023). Twelve 6-week-old male mice were randomized into four groups (n=3) and intravenously administered Mn3O4, DNase-1, MD, or MD@NM. On day 7, blood samples were analyzed using a Sysmex XT-2000i hematology analyzer (Kobe, Japan), and major organs were collected for H&E staining and histopathological assessment using an Olympus DX51 microscope (Tokyo, Japan).

For biodistribution, thirty-six 6-week-old male C57BL/6 mice were divided into six groups (n=3). Cy5-labeled MD@NM was administered intravenously, and major organs (heart, liver, spleen, lung, kidney) were harvested at 0.5, 2, 4, 6, and 12 h post-injection. Fluorescence distribution was visualized using an IVIS Spectrum imaging system (PerkinElmer, Shanghai, China).

**LPS-Induced SAKI Mouse Model**

Twelve 6-week-old male C57BL/6 mice were divided into four groups (n=3) and subjected to LPS-induced SAKI. Two hours post-LPS, different nanozymes formulations were administered intravenously to evaluate therapeutic efficacy; untreated mice served as controls. Renal function (BUN and CRE), H&E/PAS histology, and TUNEL immunofluorescence staining were assessed 24 h post-treatment. Blood samples were collected in heparinized tubes and centrifuged (2,000 × g, 15 min, 4 °C) for serum biochemical and cytokine analyses.For histology, kidneys were fixed in 4% paraformaldehyde, paraffin-embedded, sectioned, and stained (H&E, PAS, BAX IHC, TUNEL). Frozen tissues were sectioned (5 μm) for ROS detection using dihydroethidium (DHE; 10 μM, 30 min) as a redox-sensitive probe. Sections were imaged via confocal microscopy (Nikon A1RS, Nikon Instruments).

**Transcriptome analysis flow**

*Quality control:* Samples are sequenced on the platform to get image files, which are transformed by the software of the sequencing platform, and the original data in FASTQ format (Raw Data) is generated. Sequencing data contains a number of connectors, low-quality Reads, so we use Cutadapt (v1.15) software to filter the sequencing data to get high quality sequence（Clean Data）for further analysis .

*Reads mapping:* The reference genome and gene annotation files were downloaded from genome website. The filtered reads were mapping to the reference genome using HISAT2 v2.0.5.

*Differential expression analysis:* we used HTSeq (0.9.1) statistics to compare the Read Count values on each gene as the original expression of the gene, and then used FPKM to standardize the expression. Then difference expression of genes was analyzed by DESeq (1.30.0) with screened conditions as follows: expression difference multiple |log2FoldChange| > 1, significant P-value < 0.05. At the same time, We used R language Pheatmap (1.0.8) software package to perform bi-directional clustering analysis of all different genes of samples. We geted heatmap according to the expression level of the same gene in different samples and the expression patterns of different genes in the same sample with Euclidean method to calculate the distance and Complete Linkage method to cluster.

*GO and KEGG enrichment analysis:* we mapped all the genes to Terms in the Gene Ontology database and calculated the numbers of differentially enriched genes in each Term. Using topGO to perform GO enrichment analysis on the differential genes, calculate P-value by hypergeometric distribution method (the standard of significant enrichment is P-value <0.05), and find the GO term with significantly enriched differential genes to determine the main biological functions performed by differential genes. ClusterProfiler (3.4.4) software was used to carry out the enrichment analysis of the KEGG pathway of differential genes, focusing on the significant enrichment pathway with P-value <0.05.

*New transcript analysis:* On the basis of the existing reference genome, using the software StringTie (http://ccb.jhu.edu/software/stringtie/) to assemble the mapped reads, and comparing the splicing results with the known transcripts to obtain no annotations Transcripts of information.

*Differential variable shear analysis:* Using rMATS (3.2.5) software to analyze differential variable shear events. The main types of variable shear events analyzed are mainly SE, RI, MXE, A5SS, A3SS.

*SNP and indel analysis:* The Varscan program was used to obtain SNP and InDel sites, and the filtering criteria were:

1) SNP site base Q>20;

2) The number of Reads covering the site> 8;

3) The number of Reads supporting the mutation site> 2;

4) The p-value of SNP locus is <0.01. Analysis of variant sites.

*Transcription factor family analysis:* The prediction of transcription factors is to compare plants and animals with PlantTFDB (Plant Transcription Factor Database) and AnimalTFDB (Animal Transcription Factor DataBase) databases respectively to predict the transcription factor and the family information to which the transcription factor belongs.

*Exon difference analysis:* Using the DEXSeq package to analyze the difference in exon usage in RNA-seq experimental data, where the difference in exon usage refers to the relatively different exon usage due to experimental conditions.

*Interaction Analysis of Differential Gene Protein Network:* The STRING database (https://string-db.org/) is used for protein interaction analysis to reveal the relationship between target genes.

**Data analysis**

All the experiments were repeated at least three times. Student’s unpaired or paired t-tests were used to analyze the significance of the differences between two groups with GraphPad Prism 9.5 software (GraphPad Software, San Diego, CA). We used unpaired multiple t-tests and analysis of variance (ANOVA) to analyze differences among multiple groups. The statistical tests were two-sided, and values of p < 0.05 were considered statistically significant.

**
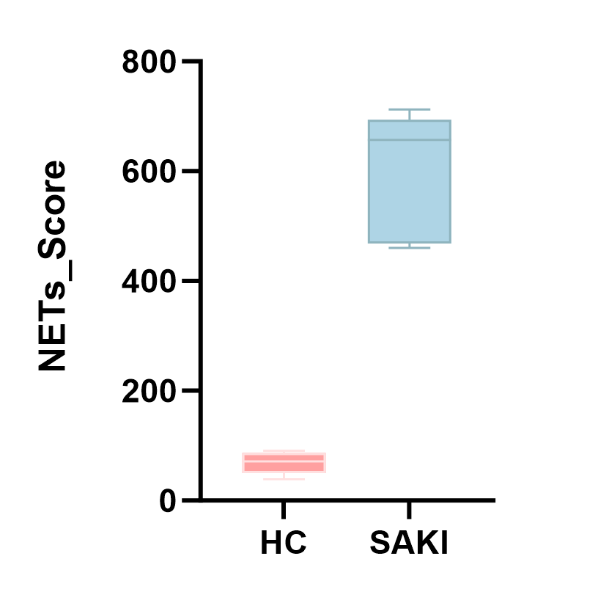
**

**Figure S1**. NETs activity score in healthy controls and SAKI patients. The data are presented as the means ± SD (n = 3).


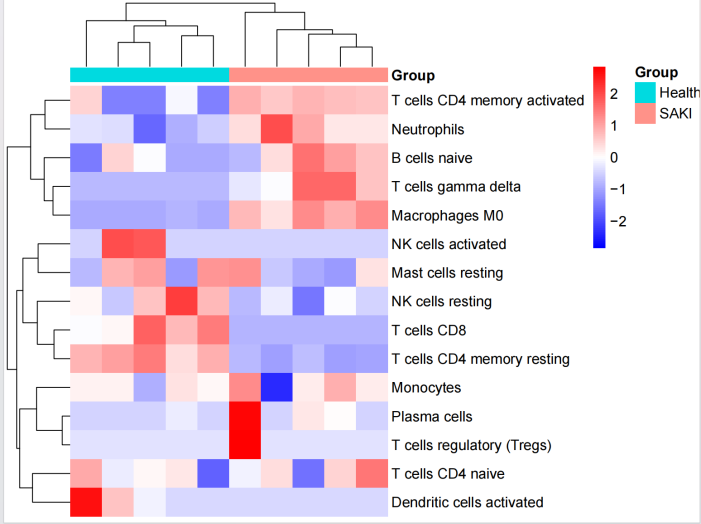


**Figure S2.** Neutrophil activation in healthy controls and SAKI patients.


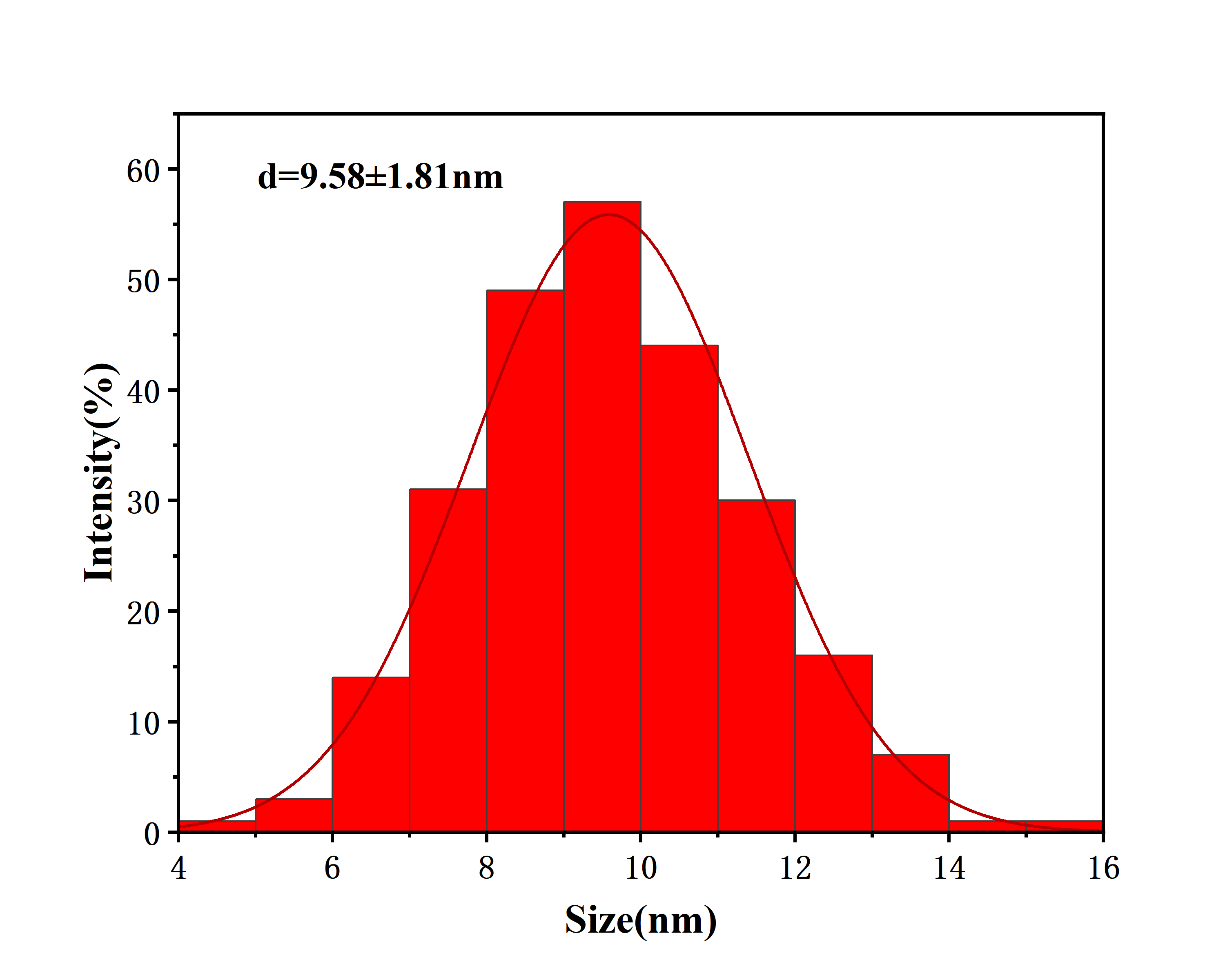


**Figure S3.** Size distributions of Mn_3_O_4_. The data are presented as the means ± SD (n = 3).
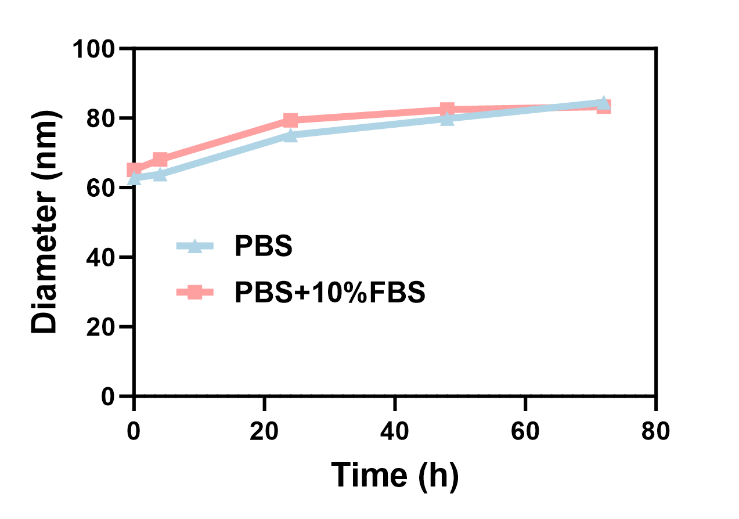


**Figure S4.** DLS size distribution of MD@NM in PBS and 10% FBS. The data are presented as the means ± SD (n = 3).


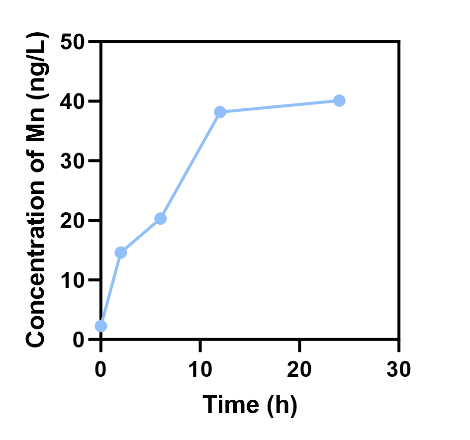

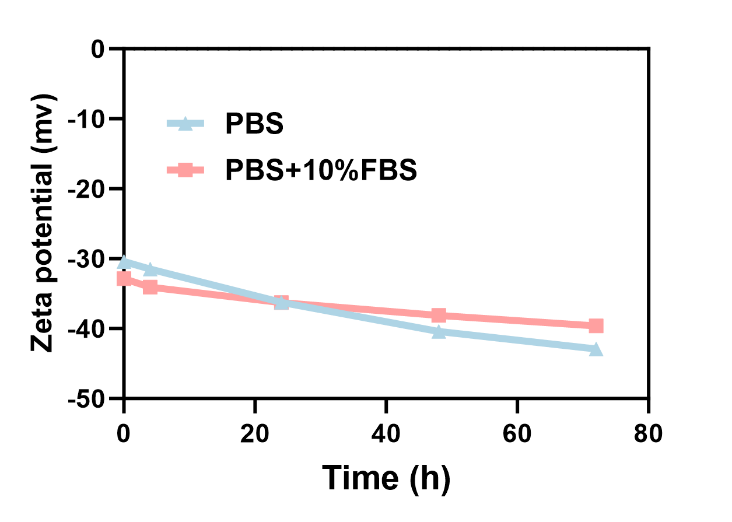
**Figure S5.** Zeta potential of MD@NM after incubation in PBS and 10% FBS. The data are presented as the means ± SD (n = 3).

**Figure S6.** Evaluation of ionic stability and Mn^2+^ leakage from Mn_3_O_4_-based nanoscavengers under physiological conditions. The data are presented as the means ± SD (n = 3).


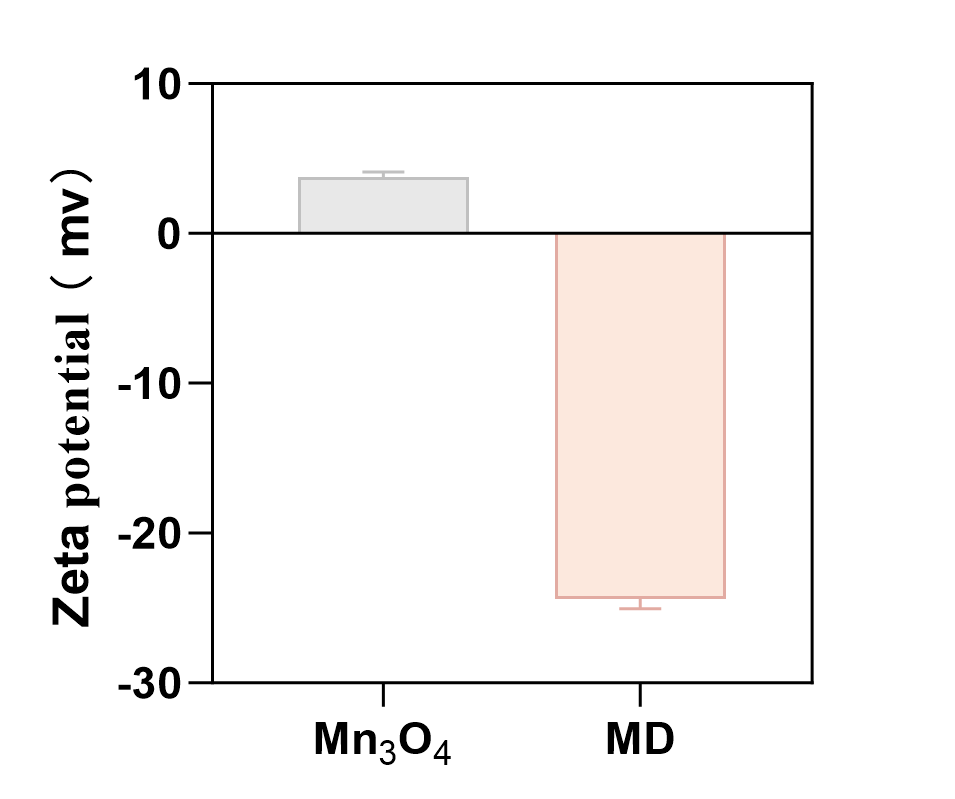


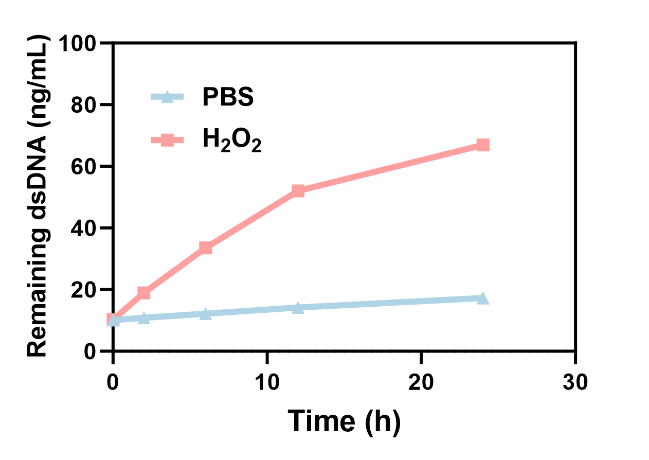
**Figure S7.** Zeta potentials of Mn_3_O_4_ and MD. The data are presented as the means ± SD (n = 3).

**Figure S8.** Enzymatic activity and oxidative stability of DNase-1 integrated in MD@NM. The data are presented as the means ± SD (n = 3).


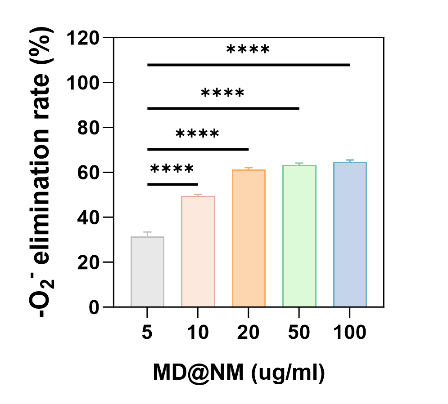

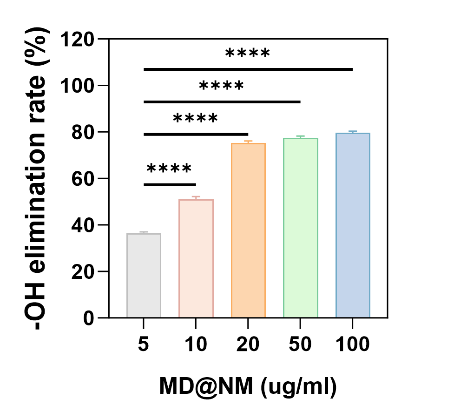
**Figure S9.** SOD-like activity of MD@NM at different concentrations. The data are presented as the means ± SD (n = 3). Statistical significance was determined by ANOVA. *p < 0.05, **p < 0.01, ***p < 0.001; ns, not significant.


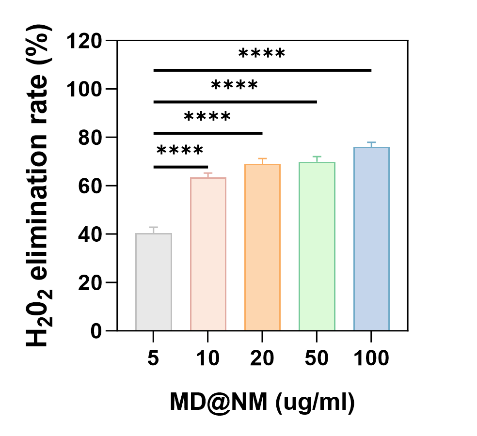
**Figure S10.** MB degradation assay of MD@NM. The data are presented as the means ± SD (n = 3). Statistical significance was determined by ANOVA. *p < 0.05, **p < 0.01, ***p < 0.001; ns, not significant.

**Figure S11.** CAT-like activity of MD@NM at different concentrations. The data are presented as the means ± SD (n = 3). Statistical significance was determined by ANOVA. *p < 0.05, **p < 0.01, ***p < 0.001; ns, not significant.


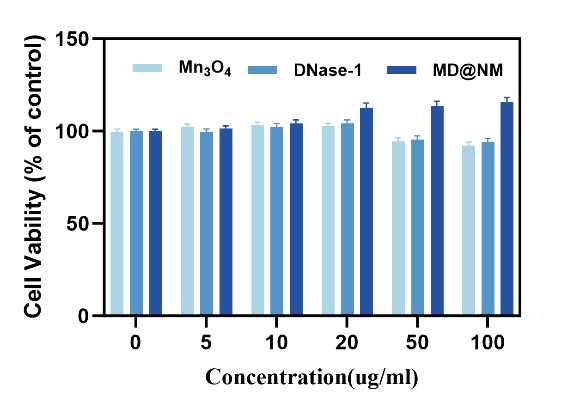


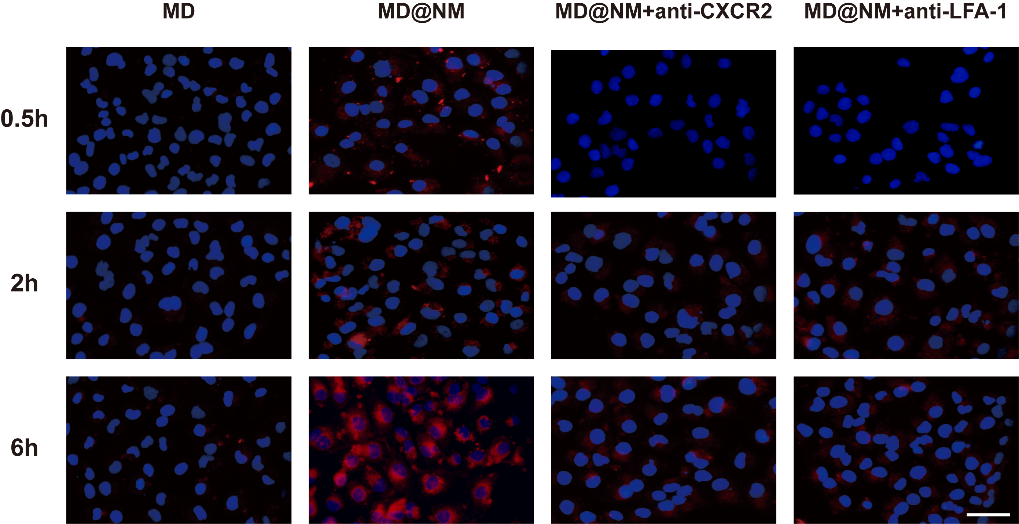
**Figure S12.** Cell viability under various concentrations of MD@NM treatment in macrophages. The data are presented as the means ± SD (n = 3).

**Figure S13.** Quantitative analysis of fluorescence uptake after CXCR2 and LFA-1 blocking. Scale bar: 100 μm


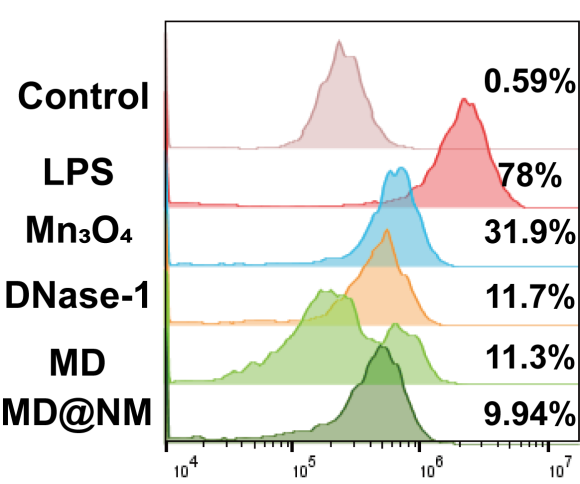


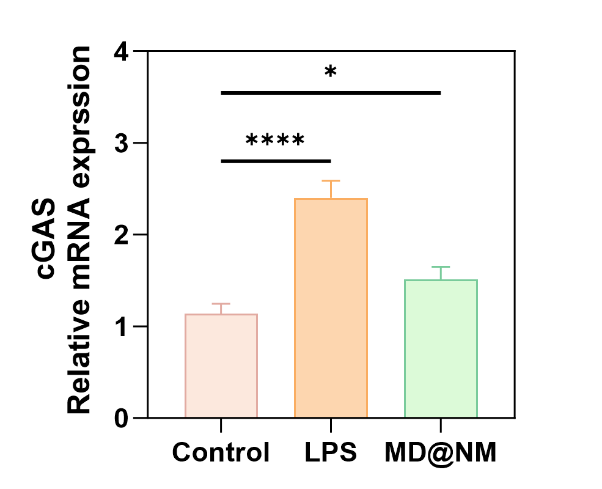
**Figure S14.** FCM of ROS analysis.

**Figure S15.** qPCR analysis of cGAS mRNA expression. The data are presented as the means ± SD (n = 3). Statistical significance was determined by ANOVA. *p < 0.05, **p < 0.01, ***p < 0.001; ns, not significant.


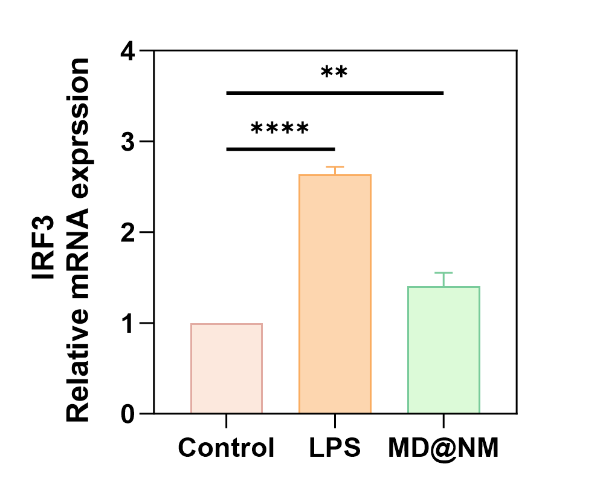

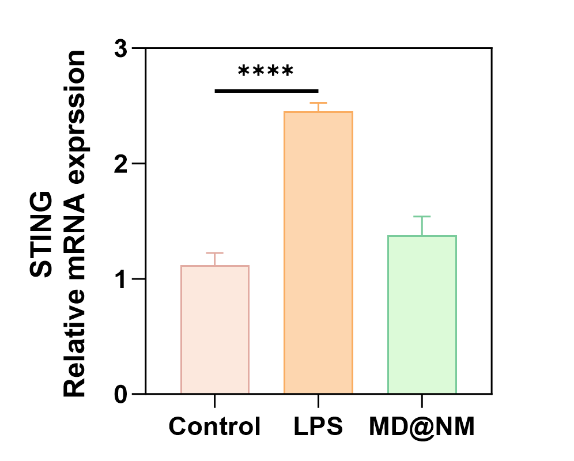
**Figure S16.** qPCR analysis of STING mRNA expression. The data are presented as the means ± SD (n = 3). Statistical significance was determined by ANOVA. *p < 0.05, **p < 0.01, ***p < 0.001; ns, not significant.

**
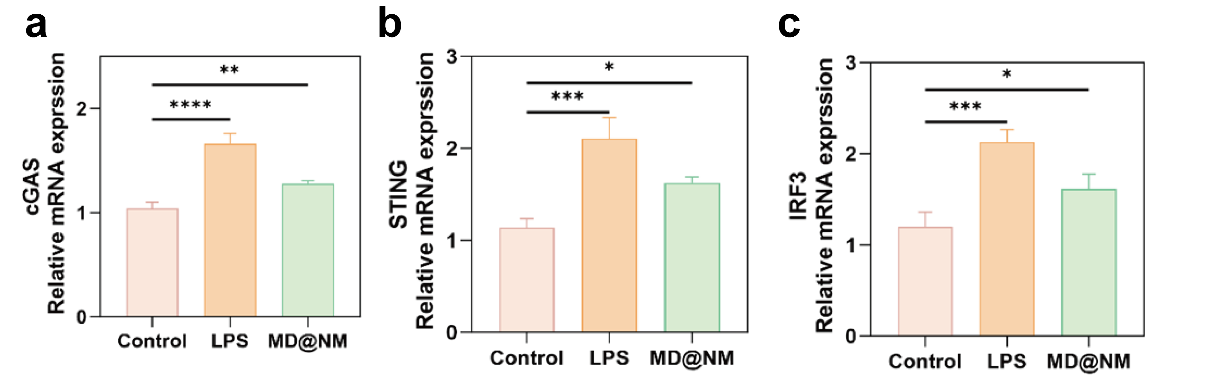
Figure S17.** qPCR analysis of IRF3 mRNA expression. The data are presented as the means ± SD (n = 3). Statistical significance was determined by ANOVA. *p < 0.05, **p < 0.01, ***p < 0.001; ns, not significant.

**
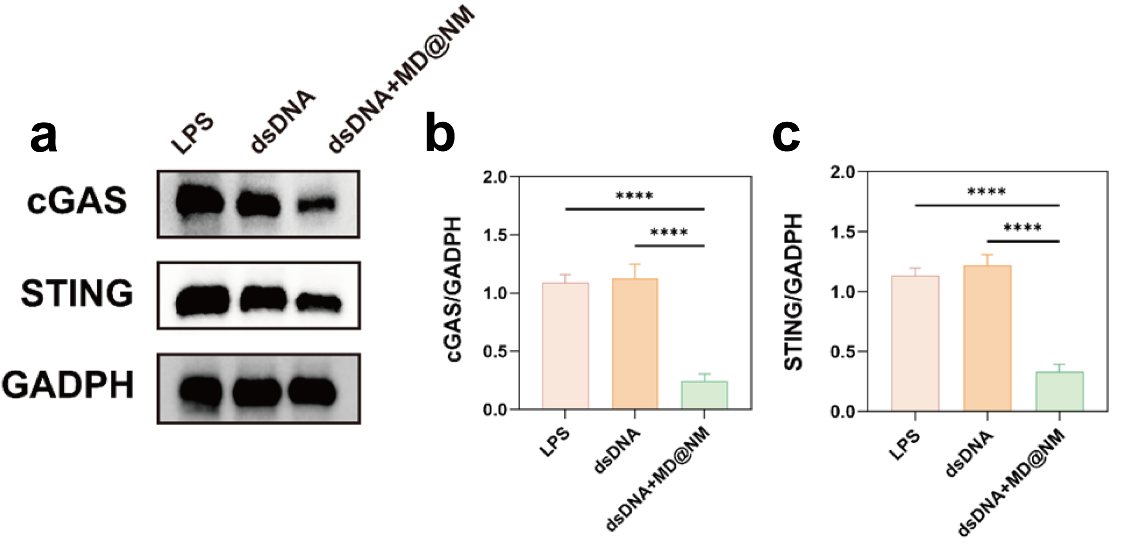
Figure S18.** qPCR validation of cGAS (a), STING (b), IRF3 (c) expression in renal tissues. The data are presented as the means ± SD (n = 3). Statistical significance was determined by ANOVA. *p < 0.05, **p < 0.01, ***p < 0.001; ns, not significant.

**Figure S19.** Schematic illustration of dsDNA-mediated rescue of the cGAS-STING pathway. The data are presented as the means ± SD (n = 3). (a) Representative Western blots of cGAS and STING (b) Quantitative densitometric analysis of cGAS protein levels normalized to GAPDH. (c) Quantitative densitometric analysis of STING protein levels normalized to GAPDH. Statistical significance was determined by ANOVA. *p < 0.05, **p < 0.01, ***p < 0.001; ns, not significant.


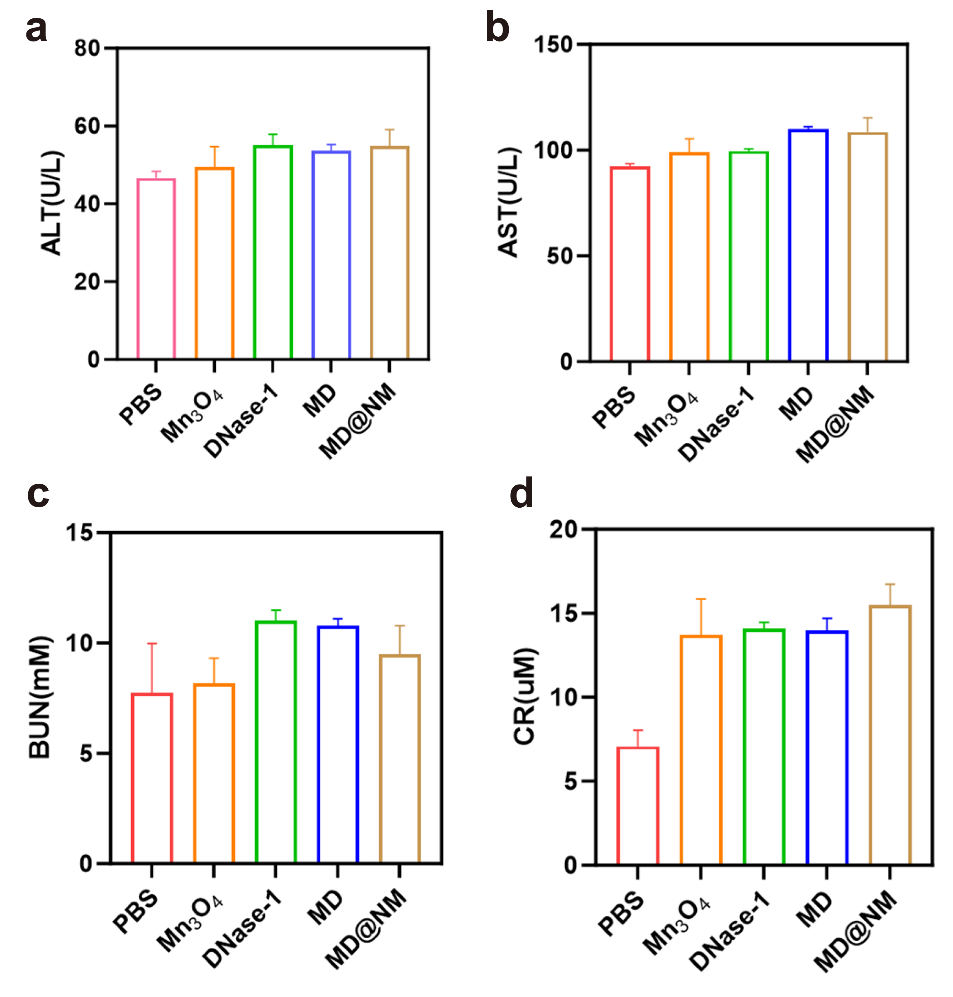

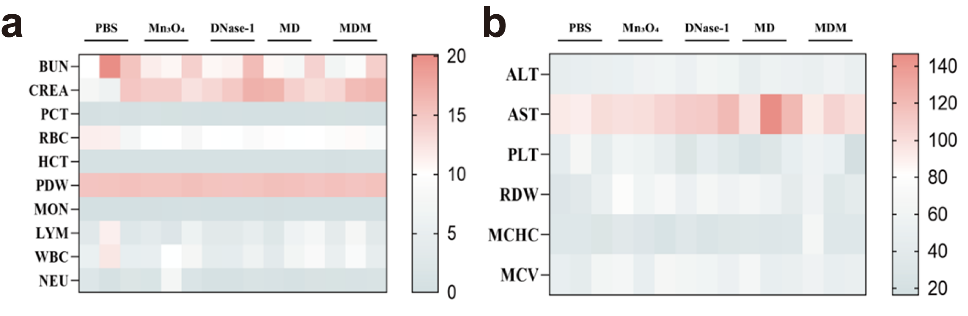
**Figure S20.** Blood biochemistry and hematology analysis of C57BL/6J mice after various treatments. The data are presented as the means ± SD (n = 3).

**Figure S21.** Complete blood count tests of mice after various treatments. The levels of alanine aminotransferase (ALT) (a), aspartate aminotransferase (AST) (b), blood urea nitrogen (BUN) (c) and creatinine (CR) (d) in the blood of mice after various treatments. Data are presented as means ± S.D. (n = 3).


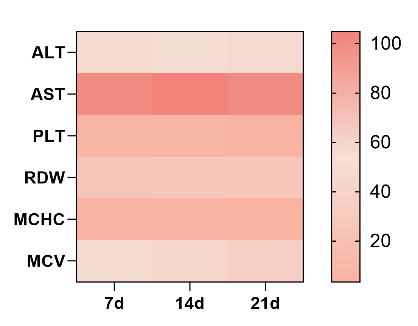

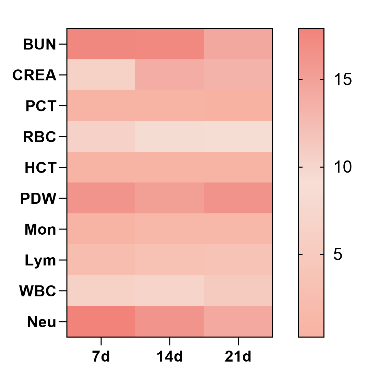

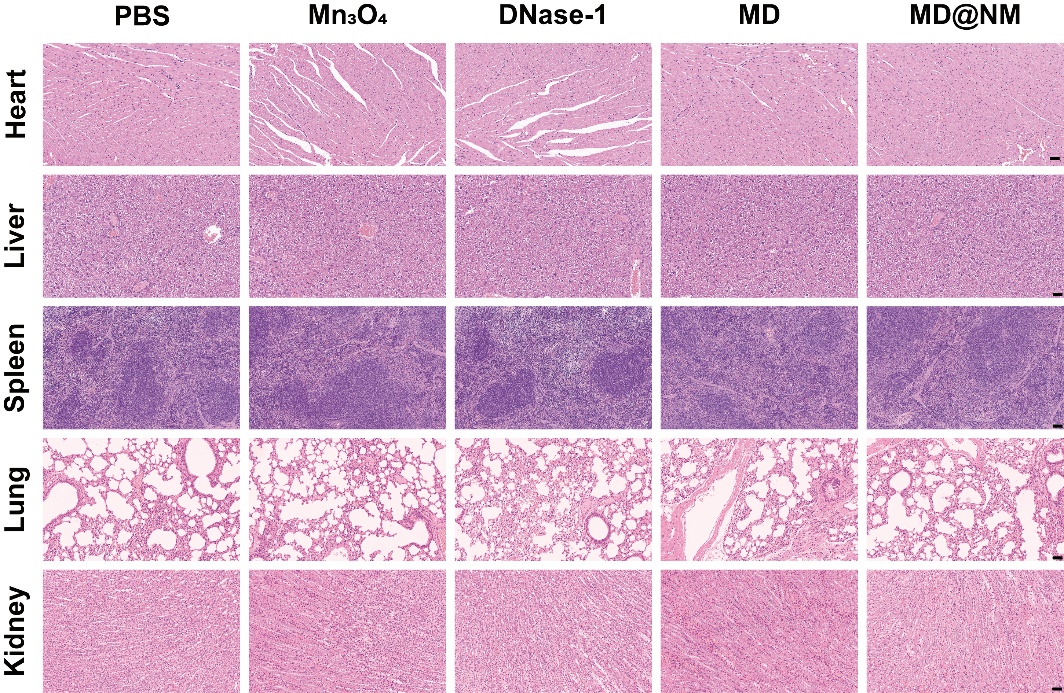
**Figure S22.** H&E staining of major organs from the mice after various treatments. Scale bar: 50 μm

**Figure S23.** Blood biochemistry and hematology analysis of C57BL/6J mice at 7, 14, and 21 days post-injection.

**
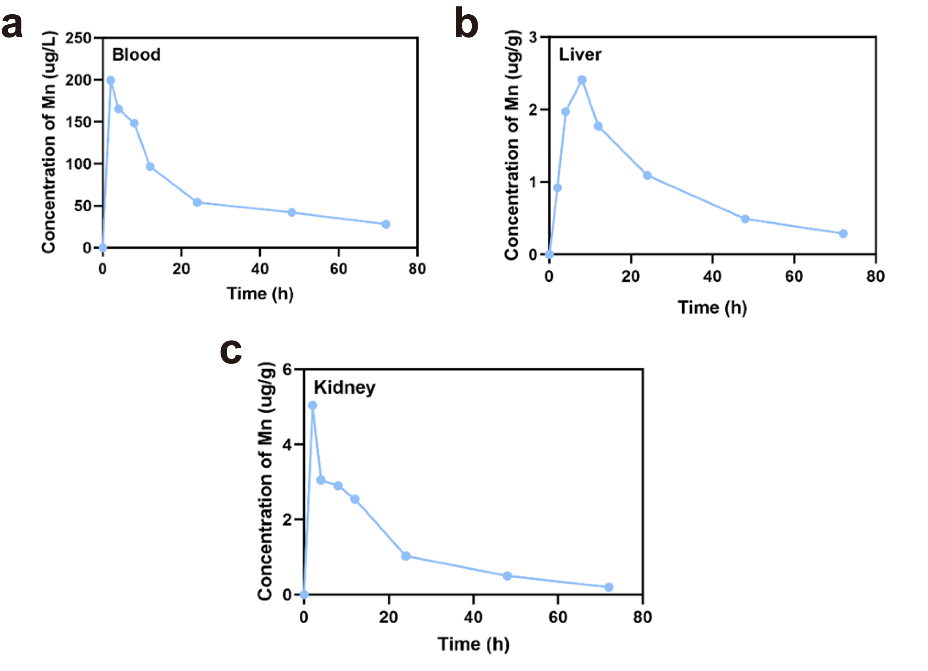

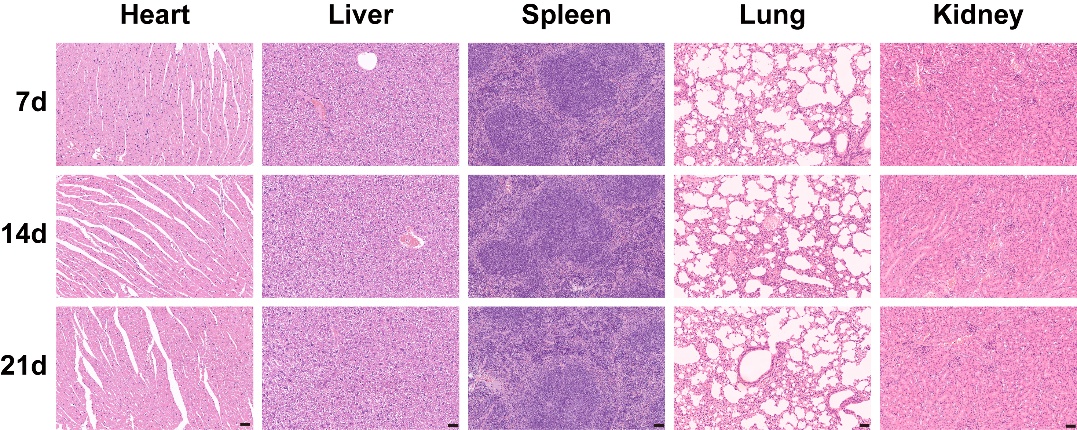
Figure S24.** H&E staining of heart, liver, spleen, lung, and kidney at 7, 14, and 21 days post-injection. Scale bar: 50 μm


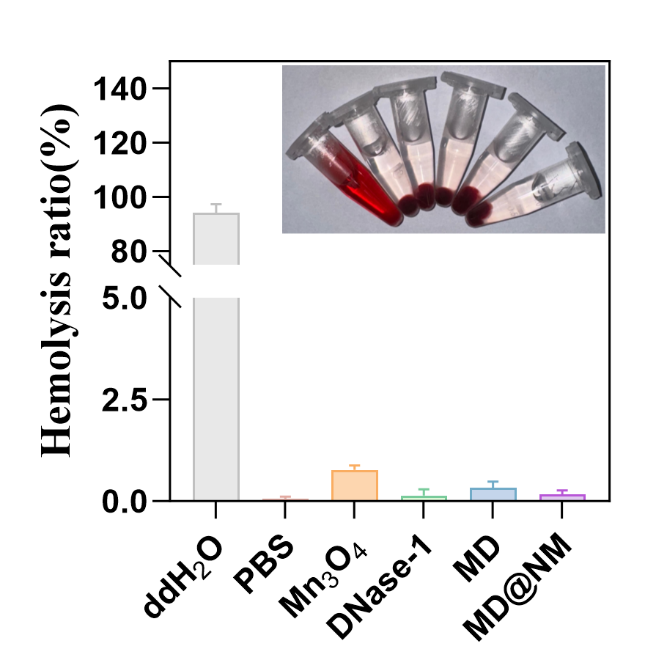
**Figure S25.** Time-dependent manganese (Mn) concentrations in (a) blood, (b) liver, and (c) kidney after intravenous administration of MD@NM were quantified by inductively coupled plasma mass spectrometry (ICP-MS). The data are presented as the means ± SD (n = 3).

**Figure S26.** The representative digital photos of hemolysis tests using mouse red blood cells

and the corresponding quantitative analysis. The data are presented as the means ± SD (n = 3).
